# Optimized multichannel 4 mA vs conventional transcranial direct current stimulation for major depressive disorder: A randomized sham-controlled trial

**DOI:** 10.1038/s41380-026-03560-0

**Published:** 2026-04-02

**Authors:** Mohammad Ali Salehinejad, Marzieh Abdi, Mohsen Dadashi, Maryam Habibi, Amir-Homayun Hallajian, Kiomars Sharifi, Ali Khadem, Ricardo Salvador, Giulio Ruffini, Michael A. Nitsche

**Affiliations:** 1 Department of Child and Adolescent Psychiatry, Psychosomatics and Psychotherapy, Medical Faculty, RWTH Aachen University, Aachen, Germany; 2 Department of Psychology and Neurosciences, Leibniz Research Centre for Working Environment and Human Factors, Dortmund, Germany; 3 Department of Clinical Psychology, Faculty of Medicine, Zanjan University of Medical Sciences, Zanjan, Iran; 4 Donders Institute for Brain, Cognition, and Behaviour, Radboud University, Nijmegen, The Netherlands; 5 Department of Information Technology and Electrical Engineering, ETH Zurich, Zurich, Switzerland; 6 Department of Biomedical Engineering, Faculty of Electrical Engineering, K. N. Toosi University of Technology, Tehran, Iran; 7 Neuroelectrics Barcelona, Barcelona, Spain; 8 Bielefeld University, University Hospital OWL, Protestant Hospital of Bethel Foundation, University Clinic of Psychiatry and Psychotherapy, Bielefeld, Germany; 9 German Center for Mental Health, Bochum, Germany

**Keywords:** Depression, Neuroscience

## Abstract

Major depressive disorder is a prevalent mental condition and the second most disabling disease. Transcranial direct current stimulation (tDCS), with advantages such as affordability, home-use application, and mild side effects, has been proposed for treating depression. Yet, its clinical efficacy is not established. Optimizing tDCS interventions for more effective and feasible clinical application is a need and topic of ongoing research. This randomized, sham‑controlled trial compares the efficacy of computationally optimized multichannel tDCS (4 mA), a less source-demanding alternative for personalized tDCS, vs conventional tDCS (2 mA) for treating unipolar depression. Seventy-one patients were randomly assigned to the optimized multichannel tDCS, conventional tDCS, and sham tDCS arms, of whom 60 provided endpoint data. In the optimized multichannel tDCS, electrical current was delivered via 7 small electrodes (max 1.65 mA per electrode; total injected current per polarity ≈ 4 mA [precisely 3.99 mA]) while in the conventional (2 mA per electrode) and sham tDCS, two 35 cm² sponge electrodes were used. Intervention efficacy and treatment response were evaluated before the intervention, at weeks 2, 4, 6, and 1- and 3-month post-intervention, and cognitive functions and brain connectivity changes were assessed before and after the intervention. Both active tDCS interventions significantly reduced depressive symptoms compared to the sham group after the intervention. The optimized multichannel tDCS demonstrated earlier and stronger symptom alleviation than conventional tDCS. It additionally improved cognitive control and modulated functional connectivity markers associated with depression pathophysiology. The mean change in the primary clinical outcome from baseline to the study endpoint was 6.30 in the sham group and 13.50 and 21.50 in the conventional and multichannel tDCS groups, with corresponding response rate (≥50% symptom reduction) of 20% (4/20), 45% (9/20), and 75% (15/20), respectively. Blinding was unsuccessful only in the optimized multichannel arm, likely due to more intense stimulation-related sensations, however, this did not impact clinical outcomes. The optimized multichannel 4 mA tDCS shows clinical efficacy for treating depression, warranting further investigations in the future. Trial Registration Identifier: NCT06165445 / IRCT20210517051330N1.

## Introduction

Major depressive disorder (MDD) is the most common mental health disorder and the second most disabling disease, according to the latest Global Burden of Disease report [1, 2]. Antidepressants, especially Selective Serotonin Reuptake Inhibitors (SSRIs), and psychotherapeutic interventions, especially cognitive-behavioral therapy, are considered first-line treatments for MDD with severe and mild to moderate severity [3, 4]. These treatments, nevertheless, come with some limitations. For example, SSRIs have delayed therapeutic benefits, taking about 4 to 6 weeks to ameliorate symptoms [5] and while psychotherapies are effective compared to control conditions, the absolute response rates are modest [6]. Accordingly, developing alternative therapeutic interventions that overcome these limitations and are guided by current knowledge of MDD pathophysiology remain an active field of research.

No established mechanism can explain all aspects of MDD. However, previous neuroimaging studies have shown that MDD is associated with functional changes in brain circuits such as the cognitive control and affective salience networks [7–9]. Furthermore, resting state electroencephalography (EEG) studies have shown that depression is associated with reduced or altered neural synchronization [10, 11] and lower coherence [11, 12] within the default mode and central executive networks. These altered brain functions/networks provide opportunities for developing novel interventions that aim to more specifically restore impaired functional changes in MDD, such as noninvasive brain stimulation (NIBS) techniques [13–15]. Transcranial direct current stimulation (tDCS) is a NIBS technique that uses weak direct electrical current to modulate brain excitability [16, 17]. tDCS devices offer a portable, low-cost, safe and user-friendly brain stimulation treatment [18, 19] suitable for home use and self-administration, making them a potentially ideal first-line, noninvasive option before considering more expensive and less accessible interventions like transcranial magnetic stimulation and electroconvulsive therapy [20].

In contrast to a recent multi-center trial that did not find tDCS superior for treating MDD [21], a more recent multi-center study showed that a 10-week home-based tDCS treatment in MDD has high efficacy, acceptability, and safety [22]. While this supports the potential of tDCS for treating MDD, it also underscores the need for more well-powered trials and/or optimized interventions. A continued line of research in NIBS clinical application is to optimize and personalize tES intervention to enhance clinical efficacy [23]. While individualized NIBS intervention based on patient-specific brain imaging shows promise [24–26] its complexity and resource demands limit widespread clinical use. In contrast, tDCS offers a practical alternative through multichannel configurations that can be optimized by computational modelling to more effectively modulate the left and right prefrontal cortices, the main stimulation target in depression [27–29]. With computational modeling of electrical field flow, a multichannel tDCS can target specific brain regions more effectively across individuals, balancing precision with accessibility [30]. This approach avoids the practical barriers of individualized imaging required for personalized tDCS [26], while still improving modulation of MDD-related brain networks compared to conventional tDCS montages.

**Fig. 1:**
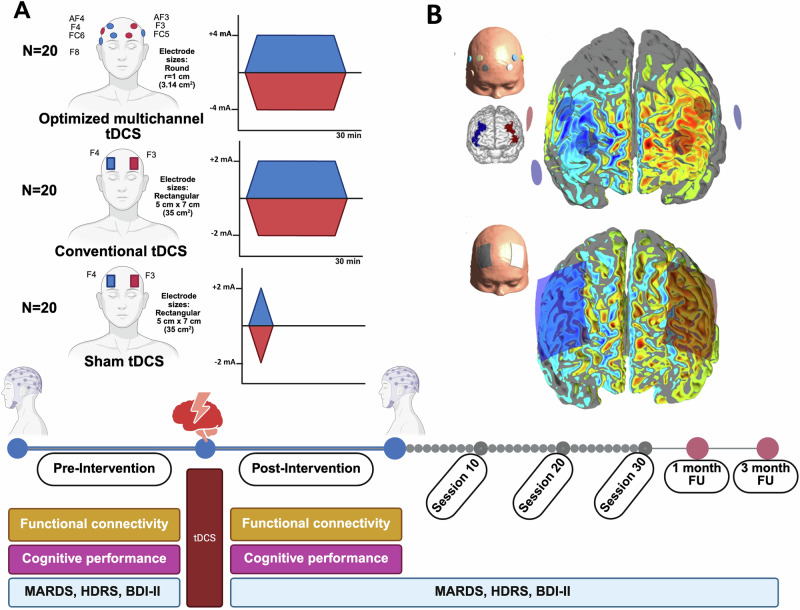
Study design, tDCS montages and E‑field distribution. **A**, Study procedure. The experiment was conducted in a randomized, double-blind, sham-controlled parallel-group design. Patients were assigned to one of three tDCS groups: 2-mA conventional tDCS (n = 20), 4-mA optimized multichannel tDCS (n = 20), and sham tDCS (n = 20). All participants underwent 30 daily stimulation sessions. Depressive symptoms were evaluated before the intervention, at weeks 2 (sesson 10), 4 (session 20), 6 (session 30), and up to one and three months follow-up. Cognitive control performance and resting-EEG functional connectivity were assessed before and after the intervention. **B**, Distribution of the normal component of the E-field (En, in V/m) in a surface inside the grey matter tissue, between the grey matter-CSF and white matter-grey matter interfaces. The bottom panel shows the En-distribution for the conventional protocol using a bipolar electrode montage with rectangular 7×5 cm sponge electrodes (anode/cathode over F3/F4) at +2/-2-mA intensity (current density = 0.057 mA/cm²). The top panel illustrates the En-distribution for the optimized multichannel protocol using 7 small sponge electrodes (with a radius of 1.0 cm and a contact area of ≈3.14 cm^2^, anodes shown in red, cathodes in blue) targeting Brodmann area 46, intended to enhance and reduce excitability in the left and right dorsolateral prefrontal cortices, respectively. In the optimized multichannel stimulation condition, three anodes over the F3 (1.10 mA, current density ≈0.35 mA/cm²), AF3 (1.24 mA, current density ≈0.40 mA/cm²), and FC6 (1.65 mA, current density ≈0.53 mA/cm²) and four cathodes over the F4 (-1.28 mA, current density ≈0.41 mA/cm²), AF4 (-1.11 mA, current density ≈0.35 mA/cm²), F8 (-0.62 mA, current density ≈0.20 mA/cm²), and FC5 (-0.97 mA, current density ≈0.31 mA/cm²) were included for anodal and cathodal stimulation respectively. Positive En values indicate an inward-directed field, corresponding to increased cortical excitability (expected anodal effect), whereas negative En values indicate the opposite direction and reduced excitability (expected cathodal effect), according to the lambda-E model. Figure 1B shows that the target regions in the left and right prefrontal cortices are modulated more homogeneously in the optimized multichannel tDCS than in the conventional tDCS.

In this registered randomized, sham-controlled, double-blind trial, we evaluated the clinical efficacy and safety of an optimized multichannel 4 mA (precisely 3.99 mA) tDCS protocol (using seven small electrodes, 1.65 mA per electrode) and a conventional 2 mA tDCS (two 35 cm² electrodes, 2 mA per electrode) compared to sham tDCS. Over 30 daily sessions, we carefully monitored treatment response and symptom changes in five time points during and after the intervention and conducted follow-up assessments at 1 and 3 months. We also investigated how these tDCS interventions affect cognitive control and brain functional connectivity right after the intervention (secondary outcome measures). Figure 1 illustrates the study design and interventions. To our knowledge, this study is the first randomized sham-controlled trial to examine the impact of a computationally optimized multichannel tDCS for MDD treatment head‑to‑head against both conventional and sham tDCS, and the first tDCS trial in depression to apply a 4 mA total injected current (precisely 3.99 mA). We expected therapeutic efficacy of active interventions compared to sham, and aimed to explore whether and how the interventions differ in efficacy, safety, and blinding among the active interventions.

## Methods

### Participants

This was a randomized, double-blind, parallel-group study. The study was prospectively registered before data collection (ID: IRCT20210517051330N1) and later also on ClinicalTrials.gov (ID: NCT06165445). We included patients with unipolar, nonpsychotic MDD per DSM-5 criteria, confirmed by psychiatrists using the Mini-International Neuropsychiatric Interview (MINI). The CONSORT flowchart of study inclusion is displayed in Figure S1 (supplementary content). Of 167 screened patients, 87 were assessed for eligibility across several neuropsychiatric clinics in the city of Zanjan from 2021-2023. Sample size was calculated using power analysis based on a small-to-medium effect size (f = 0.20 equivalent to *η*p2 = 0.04, α = 0.05, power=0.95, mixed-model ANOVA, *N* = 57). We increased the sample size to 60 subjects to compensate for an anticipated 5% dropout rate. In addition, a sample size re-estimation was prespecified and performed to address the dropout rate in case of more than expected dropouts (supplementary content). At the end, a total of 71 patients were randomized; of these, 60 completed the intervention and provided endpoint and follow-up data (n = 20 per arm). The number of randomized participants and dropouts per arm will be reported in the CONSORT flow diagram and ITT analysis (see Figure S1). Attrition rates were 2 (multichannel), 4 (conventional), and 5 (sham). The inclusion criteria were: (1) being 18–60 years of age, (2) failure with at least one antidepressant medication, (3) not currently using antidepressants or having a stable treatment regimen for at least 6 weeks before and throughout the experiment, and (4) unipolar depression diagnosis. Patients had to have mild to severe depression according to the Hamilton Depression Rating Scale, as well as a low risk of suicide (evaluated based on the MINI). Exclusion criteria were other Axis I disorders, including alcohol or substance use or dependence (anxiety disorders comorbidity was allowed), any Axis II disorders, previous neurological conditions, and specific contraindications for tDCS (e.g., metallic plates in the head). All participants were native speakers and had normal or corrected-to-normal vision. This registered clinical trial was approved by the Ethics Committee of the Zanjan University of Medical Sciences (IR.ZUMS.REC.1400.059). All methods were performed in accordance with the ethical guidelines and regulations and in compliance with the latest Declaration of Helsinki. Participants provided written informed consent after receiving a complete description of the study. Patients’ demographics are shown in Table 1.

**Table 1:** Demographic data.

Variable		Optimized multichannel tDCS	conventional tDCS	sham tDCS	*p* *
Sample size (*n*)		20	20	20	-
Age – Mean (SD)		32.15 (12.18)	35.75 (11.38)	29.60 (8.06)	0.197
Sex – Male (female)		11 (9)	11 (9)	5 (15)	0.089
Job	student	7	7	7	0.241
	self-employed	5	5	5	
	housewife/husband	3	3	3	
	employee	4	4	4	
	unemployed	1	1	1	
Education	below diploma	1	5	0	0.047
	diploma	10	7	4	
	bachelor’s degree	6	4	11	
	master’s degree	1	2	5	
	PhD level	2	2	0	
Marital status	single	9	11	11	0.457
	married	10	9	9	
	divorced	1	0	0	
On medication (n)		17	18	15	0.368
Type of medication	SSRI	14	16	13	0.853
	Tricyclic	3	2	2	
	Other	0	0	0	

*Note*: tDCS = transcranial Direct Current Stimulation; M = Mean; SD = Standard Deviation. * = between-group differences in demographic variables were explored by Chi-square test or Fisher’s exact test for categorical variables and *F* tests for continuous variables.

### Randomization and blinding

A random allocation sequence table was generated using an online block randomization website (https://www.sealedenvelope.com/simple-randomiser/v1/lists) for three treatment groups, with a block size of 6 and 10 blocks planned to cover 60 participants. The enroller was blinded to the group allocation, and participant allocation was concealed through a central coordinator who generated and held the randomization sequence. Patients and intervention codes were transferred to the patient’s enroller by the coordinator (via phone call) while the enroller had no access to the block sequence and group assignment. The coordinator had no involvement in the actual study procedure and was merely responsible for randomization coordination. Clinical outcome measures were assessed by a licensed psychiatrist from the hospital, who had no involvement in the study, blinded to the group assignment. An independent and blinded investigator was responsible for evaluating cognitive functions and functional connectivity. To prevent potential bias from unintentional disclosure, patients in each group were kept unaware of one another, ensuring that differences in intervention setups (multichannel cap vs. conventional/sham tDCS) did not compromise blinding.

### Primary and secondary outcome measures

The primary outcome measures to examine the effects of the interventions on depressive symptoms were the change in depressive symptoms measured by the Montgomery–Åsberg Depression Rating Scale (MADRS) [31] and Hamilton Depression Rating Scale (HDRS) score [32] from the baseline. The Beck Depression Inventory (BDI-II) [33], a self-report questionnaire in contrast to clinician-administered HDRS and MADRS, was also used as the secondary outcome measure. Response was defined a priori as a ≥ 50% reduction from baseline on MADRS/HDRS. A detailed description of these measures is in the supplementary content. The secondary measures included cognitive assessment via neuropsychological tests dedicated to depression from the CANTAB computerized test battery [34] and changes in functional brain connectivity right after the intervention. Specifically, working memory (measured by Spatial Working Memory-SWM), sustained attention (measured by Rapid Visual Processing-RVP), executive functions (measured by One Touch Stockings of Cambridge - OTS), and brain synchronization patterns (PLV, coherence) were assessed (detailed description in supplementary content).

### tDCS intervention

Table 2 provides a transparent summary of tDCS interventions, according to the RATES checklist [35]. To ensure feasibility with a high number of daily sessions per subject, two electrical stimulators were used. Each device’s performance was verified by connecting a 5 kΩ load resistor to simulate head resistance. A digital multimeter confirmed a stable 2 mA output (ranging from 1.9–2.1 mA), while an oscilloscope verified a clean DC signal free of noise or ripples. The electrode texture was the same in all stimulation conditions. In conventional tDCS, direct currents were generated by an electrical stimulator (Mind Alive, Canada) [36], applied through two saline-soaked sponge rubber electrodes (7×5 cm) for 30 minutes (with 30 s ramping up and down) and with an intensity of 2 mA per electrode (current density = 0.057 mA/cm² at 2 mA). In sham tDCS with the same device, the electrical current was ramped up and down each for 30 seconds and then turned off [37]. In the optimized multichannel protocol, stimulation was delivered with the StarStim 8 stimulator (Neuroelectrics, Spain) with an intensity of 4 mA (precisely 3.9 mA) for total injected current using 7 small round electrodes (Sponstim electrodes, radius of 1.0 cm, contact area of ≈ 3.14 cm^2^) with real-time impedance monitoring. In conventional and sham conditions, anodal and cathodal electrodes were placed over the left DLPFC (F3), and right DLPFC (F4), respectively, using the EEG 10–20 system for electrode positioning with an inter-electrode distance of at least 6 cm. Stimulation was delivered on 5 consecutive days for six weeks.

**Table 2:** RATES (Report Approval for Transcranial Electrical Stimulation) checklist.

**Participants**	Sample Size (Dx), Recruitment Process, Medication		60 out of initially 71 enrolled patients with unipolar major depression, clinic/hospital referral across Zanjan, Iran (2021-2023), stable medication regimen (n = 50 on medication)	
	Age(yrs), Sex(F:M), Edu(yrs), Handedness(R:L:D)		32.5 ± 10.81, 33:27, see Table 1, n/s	
	Hours of Sleep, Consumption of Caffeine, Nicotine, and Alcohol		Under no sleep pressure, n/s, n/s, n/s	
	Participant Eligibility Criteria: Aged 18-60 years, failure with at least one antidepressant, stable treatment regimen for at least 6 weeks (or no antidepressants), unipolar depression per DSM-5 (mild to severe per HDRS). Exclusions: other Axis I disorders (except anxiety comorbidity), any Axis II disorders, neurological conditions, tDCS contraindications (e.g., metallic plates, cardiac pacemakers, pregnancy, epilepsy). Native speakers, normal or corrected-to-normal vision.			
**Stimulator**	Stimulator: Device 1: StarStim 8 (Neuroelectrics, Spain) for multichannel tDCS, Device 2: Oasis Pro, Mind Alive (Canada)			
	Sham Option	Yes	Waveform	Constant direct current (DC)
	Output Channels	8 channels (multichannel); 2 channels (conventional/sham)	Stimulator Safety Features	Both devices had current and voltage limits
	Current Resolution	n/s	Monitoring and Feedback	In device 1 only
**Electrodes**	Positioning	International 10-20 EEG system	Inter-electrode Distance	6 cm in conventional tDCS
	Shape	Rectangular (conventional/sham); round (multichannel)	Assembly	elastic headgear in the conventional, cap in the multichannel
	Size	35 cm² (conventional/sham); ~3.14 cm² (1 cm radius, multichannel)	Contact Medium	Saline-soaked sponge
	Orientation	longitudinal along the medio-lateral axis (conventional), n/a to multichannel	Impedance	n/s
	Material	Rubber	Connector Position	n/s
	Number: Two electrodes in conventional tDCS and 7 electrodes in the multichannel tDCS			
	Montage: Conventional/sham: anode at F3 (left DLPFC), cathode at F4 (right DLPFC). Multichannel: anodes at F3 (1.10 mA), AF3 (1.24 mA), FC6 (1.65 mA); cathodes at F4 (-1.28 mA), AF4 (-1.11 mA), F8 (-0.62 mA), FC5 (-0.97 mA). Optimized via Stimweaver algorithm for excitatory left DLPFC (BA46) and inhibitory right DLPFC.			
**Current**	Intensity (mA)	2 mA	Amplitude	Peak to zero
	Density (mA/cm²)	0.057 mA/cm² (conventional); ~0.20-0.53 mA/cm² (multichannel)	Personalization	Optimized current and configuration in multichannel tDCS
	Distribution (Method)	Stimweaver algorithm in the multichannel tDCS	Duration (min)	30 min
	Frequency (Hz)	n/a	Ramp up/down (sec)	30:30
	Polarity	Anodal (left DLPFC); cathodal (right DLPFC)	Warm-up time (min)	n/a
	Waveform	Constant direct current	Sham Characteristics	30:30
**Procedure**	Study Setting and Site	Experimental lab at the hospital	Attrition (n)	11 (pre‑endpoint)
	Hypothesis Statement	Exploratory	Blinding Method	double-blinded
	Preregistration	Preregistered clinical trial	Ethical Considerations	Yes (ethics code: IR.ZUMS.REC.1400.059)
	Session Duration (min)	30 min	Safety Monitoring	yes
	Total Number of Sessions	30 sessions	Informed Consent Process	yes
	Session Frequency	5 sessions per week, (6 weeks)	Conflict of Interest	yes
	Concurrent Intervention	no	tES operator	Trained tES operator
	Randomization (Method)	yes (block randomization)	Data Analysis Plan	Mixed‑model ANOVA; modified ITT with MMRM for sensitivity/confirmation
	Counterbalancing	n/a (parallel‑group RCT)	Data Availability	yes
	Study Design: Randomized, double-blinded, parallel-group			
	Stimulation and Assessment Task Timing: Offline			
	Inter-session Interval: 1 day (multi-session design)			
	Data Collection Time Points: Post (Side-effect survey)			
	Baseline Assessment: yes			
	Control Intervention: Sham control (Initial ramp up/down stimulation only)			
	Outcome Measure: clinical measures (MADRS-HDRS-BDI-II), cognitive measures (working memory, attention, executive function), EEG (functional connectivity)			

*n/a* not applicable, *n/s* not specified, *tDCS* transcranial direct current stimulation, *HDRS* hamilton depression rating scale, *MADRS* montgomery–åsberg depression rating scale, *BDI-II* beck depression inventory-II.

The optimized multichannel stimulation involved anodal stimulation at F3 (1.10 mA, ≈0.35 mA/cm²), AF3 (1.24 mA, ≈0.40 mA/cm²), and FC6 (1.65 mA, ≈0.53 mA/cm²) and cathodal stimulation at F4 (-1.28 mA, ≈0.41 mA/cm²), AF4 (-1.11 mA, ≈0.35 mA/cm²), F8 (-0.62 mA, ≈0.20 mA/cm²), and FC5 (-0.97 mA, ≈0.31 mA/cm²) using small rubber electrodes (Fig. 1). It is of note that the maximum current density in this protocol is within the safety limit (0.60 mA/cm²) reported in a prior work [38]. In the optimized multichannel tDCS, electrode positions and currents were determined with the Stimweaver algorithm [39]. This algorithm determines the protocol (currents and electrode positions) that minimizes the weighted difference between the distribution of the normal component of the E-field in the cortical surface (En), to a target En-map. In this particular case, the target En-map was designed to stimulate the left and right DLPFC with desired excitatory and inhibitory effects, respectively. This was achieved by setting the target En as positive (inward cortical surface) on the left DLPFC region (here defined as BA46), which leads to an excitatory effect on long pyramidal cells, thus increasing excitability [39, 40]. The opposite was done for the right DLPFC target. The Stimweaver algorithm was run on a template biophysical head model [41], and the current was constrained to a maximum of 2.0 mA per electrode (in absolute value) and 3.99 mA total injected current (the sum of the currents in all the anodes and cathodes). The distribution of the component of the E-field normal to the cortical surface induced by the multichannel montage is shown in Fig. 1. This specific configuration of electrodes was expected to result in larger electric fields and more effective modulation of the target regions, according to the modeling of different electrode configurations with different intensities and electrode placements.

tDCS was applied by independent investigators who were not involved in outcome measure ratings [42]. tDCS side-effect survey was used to obtain reported side effects [43]. To minimize bias from habituation to stimulation sensations across repeated sessions, blinding efficacy was not assessed at the study endpoint. Instead, at the 3‑month follow‑up, participants indicated perceived stimulation intensity using a 3‑point scale (0=none, 1=moderate, 2=strong). Patients in the sham arm were offered an active tDCS intervention (conventional) after being informed about the sham condition. This phase was not included in the original study design.

### EEG preprocessing

EEG signals were recorded with a 21-channel EEG device with a sampling rate of 250 Hz, and the included electrodes were Fz, Cz, Pz, C3, T3, C4, T4, Fp1, Fp2, F3, F4, F7, F8, P3, P4, T5, T6, O1, O2, A1, A2. The recordings were obtained in the eyes-open condition for three minutes and then in the eyes-closed condition for three minutes (six minutes in total). To preprocess and remove artifacts from the EEG data, we used Makoto’s preprocessing pipeline in the EEGLAB 2022.1 [44] with MATLAB 2022b (The MathWorks, Natick, MA). The data were first resampled to 512 Hz to meet the software requirement, high-pass filtered at 1 Hz, and re-referenced to an average reference. We used the CleanlineNoise plugin in EEGLAB for line noise removal. We then applied Artifact Subspace Reconstruction, an automated algorithm that eliminates flatline and noisy channels, low-frequency drifts, and short-time bursts. Any removed channels were interpolated using the spherical interpolation method. Afterward, we visually inspected all the raw data to detect artifact-related parts. To remove non-brain artifacts, we applied Adaptive Mixture ICA to the EEG data to decompose independent components (ICs). We then used the EEGLAB plugin ICLabel to identify brain ICs (with a ‘brain’ label probability of more than 0.8) from all types of ICs, including Brain, Muscle, Eye, Heart, and others. Finally, we extracted a 60-s segment from the middle part of each preprocessed EEG and exported it to the BRAINSTORM software (version November 2024) for further analysis [45]. Functional connectivity was quantified using phase-locking value (PLV) and coherence across all frequency bands. A detailed description of EEG analysis is in the supplementary content. A related analysis of the same EEG data (patients and a separate healthy control group) examined directed connectivity, graph metrics, and machine learning for predicting treatment response [46]. Here, we focus on PLV/coherence contrasts between trial arms.

### Procedure

First, participants completed a brief questionnaire to evaluate their suitability for brain stimulation. All participants received 30 stimulation sessions over 6 consecutive weeks (one session per day, five days per week; Saturday–Wednesday to align with the local weekend). To avoid confounding effects of circadian factors, which can significantly affect neuroplasticity induction [47]. All stimulation sessions took place between 11:00-14:00, and participants were not under sleep pressure [48]. Clinical outcome measures were evaluated before the first intervention (pre-intervention), after the 10^th^ session, 20^th^ session, the 30^th^ session (last session- study endpoint), 1 month, and 3 months following the last stimulation session. Cognitive function assessment and EEG recordings took place only before and after the intervention. Patients were instructed about cognitive tasks before the beginning of the experiment. The study procedure is illustrated in Fig. 1A. None of the patients underwent psychotherapy at any point during the study. Participants were unaware of the study hypotheses and stimulation conditions and were offered free transportation for assessment sessions. To maintain a double-blind design, independent researchers and clinicians, blind to stimulation conditions and group assignment, evaluated the outcome measures.

### Statistical analysis

Data analyses were conducted with the statistical package SPSS, version 26.0 (IBM, SPSS, Inc., Chicago, IL), and GraphPad Prism 8.2.1 (GraphPad Software, San Diego, California). Normality and homogeneity of data distribution and variance were confirmed with the Shapiro–Wilk and Levene tests. Between‑group differences in demographic variables were explored using χ² or Fisher’s exact tests for categorical variables and *F*‑tests for continuous variables. Primary efficacy was tested with mixed model ANOVAs specifying Group (optimized multichannel tDCS, conventional tDCS, sham) as the between-subject factor and Time (pre-intervention, session 10, session 20, session 30, 1-month follow-up, 3-month follow-up; two timepoints for cognitive/EEG measures) as the within-subject factor. Sphericity was assessed with Mauchly’s test; when violated, degrees of freedom were corrected using the Greenhouse–Geisser estimator. Post hoc analyses were calculated using Bonferroni-corrected multiple comparisons for clinical and safety outcomes across groups and time points. For cognitive outcomes (SWM, RVP, OTS), pairwise contrasts were controlled with Tukey’s HSD within each mixed model ANOVA. Because these cognitive endpoints were secondary and exploratory, we did not apply additional cross-domain or cross-time multiplicity corrections.

To address attrition, a modified Intention-to-Treat (mITT) analysis including all randomized participants with baseline data (n = 71) was conducted. A mixed model for repeated measures (MMRM) was fitted with fixed effects for Group, Time, and Group×Time, a participant-level random intercept, restricted maximum likelihood estimation, and an unstructured within-participant covariance. The model incorporated all available post-baseline observations under a Missing at Random assumption (see supplementary materials). Additionally, Pearson’s correlational analyses explored associations between side effect scores/counts and clinical outcomes, and between blinding efficacy and clinical change. For binary response (≥50% reduction from baseline on MADRS, HDRS), absolute risk reduction (ARR) was computed as *p*_*t*_ *− p*_*c*_ with 95% confidence intervals (CIs) by Newcombe’s method using Wilson score limits for each proportion [49, 50]. Number needed to treat (NNT) was calculated as *1 / ARR* and reported as the ceiling to the next whole number [51]. When the ARR CI excluded 0, NNT CIs were obtained by inverting the ARR CI; when the ARR CI included 0 (which yields open‑ended bounds on the NNT scale), we reported finite two‑sided 95% Bayesian credible intervals for NNT using independent Jeffreys priors Beta for each group proportion and summarizing the posterior of *1 / p*_*t*_ *− p*_*c*_ conditional on benefit (*p*_*t*_ *˃ p*_*c*_) [52, 53].

Functional connectivity analysis was performed in Brainstorm (November 2024) [45]. Within‑group pre‑ vs. post‑intervention changes were tested using non‑parametric permutation paired t‑tests (8,000 iterations; *p* ≤ 0.05, FDR‑corrected) for each connection, and between‑group differences at baseline and post‑intervention were assessed with the same permutation framework. Results are reported as permutation‑based t‑values (FDR *p* ≤ 0.05) and visualized with a color scale spanning −4.8 to 4.8. EEG preprocessing and data analysis are detailed in the Supplementary EEG analysis.

## Results

### Baseline differences and safety outcomes

No significant differences were observed in demographic data (except for a modest difference in education- Table 1) or baseline clinical/cognitive outcomes among the three groups (Table S1). Participants tolerated the procedures well, and no serious adverse events occurred (Table S2). One-way ANOVAs indicated significant group differences across reported side effect domains, and Bonferroni‑corrected comparisons (Table S4) showed that the multichannel group reported higher itching/tingling/burning than conventional and sham (adjusted *p* ≤ 0.001); higher skin redness and headache than sham (adjusted *p* ≤ 0.001), modestly higher pain than sham (adjusted *p* = 0.049); and lower fatigue in multichannel than conventional (adjusted *p* ≤ 0.001) and sham (adjusted *p* = 0.023) tDCS. In addition to severity analyses, we quantified the *number* of side‑effect types per participant with a non‑zero average rating across all 30 sessions. The multichannel arm showed a higher side‑effect count per subject (5.05 ± 0.94) than conventional (2.50 ± 1.15) and sham (2.85 ± 1.04) (Table S5; supplementary content). Next, we did correlational analyses between the *mean* and *count* reported side effects and clinical outcomes. Despite significantly higher reported side effects in the active tDCS groups, the Pearson’s correlational analyses show that mean reported side effects generally did not correlate with the clinical outcome measures (HDRS and MADRS) (see supplementary content). The reported side-effect count, however, showed a positive correlation with symptom improvement (ΔMADRS and ΔHDRS; Pearson *r* = 0.379, *p* = 0.0028 and *r* = 0.440, *p* = 0.00044, respectively), but the association was not evident within treatment arms and was not retained after adjusting for baseline severity and group allocation (*p* ≥ 0.50). These analyses suggest that the higher side effects in the multichannel and conventional tDCS groups did not substantially affect the clinical efficacy of the treatments.

### Blinding efficacy

Blinding was evaluated at the second follow-up by asking participants to guess their treatment allocation using three response options: no stimulation (0), mild stimulation (1), or intense stimulation (2). Bang’s Blinding Index (BBI) [54] was calculated (values near 0 indicate successful blinding; positive values indicate correct-guess unblinding; negative values indicate opposite guessing). Because both active arms involved stimulation, responses of 1 or 2 were treated as “stimulation” (correct) for the multichannel and conventional groups, whereas a response of 0 was treated as correct for the sham group (stimulation vs no stimulation). Correct-guess counts were 19/20 (multichannel), 12/20 (conventional), and 4/20 (sham). The multichannel arm showed a BBI = 0.90 (95% CI 0.53 to 0.98), indicating unsuccessful blinding. The conventional arm showed BBI = 0.20 (95% CI: −0.23 to 0.56), indicating successful blinding consistent with random guessing. The sham arm showed BBI = −0.60 (95% CI − 0.84 to −0.17), indicating opposite guessing, i.e., participants in the sham condition reported “stimulation” (mild/intense) more often than “no stimulation,” despite having received sham. (supplementary Table S3).

To assess whether perceived allocation in the multichannel tDCS group could have influenced clinical response within the multichannel arm, we conducted an exploratory correlational analysis testing whether stimulation guess intensity (0–2) was associated with symptom change from baseline to the session 30 endpoint. Symptom improvement was quantified as ΔMADRS = MADRSpre − MADRS30 and ΔHDRS = HDRSpre − HDRS30, such that larger positive values indicate greater symptom reduction. In the multichannel group (n = 20), guess intensity was not correlated with symptom improvement (ΔMADRS: Spearman ρ = −0.026, *p* = 0.913; ΔHDRS: ρ = −0.062, *p* = 0.795) and was not associated with endpoint severity (MADRS30: ρ = 0.053, *p* = 0.825; HDRS30: ρ = 0.030, *p* = 0.901). A sensitivity analysis comparing participants who guessed “mild” versus “intense” stimulation was conducted using two-sided Mann–Whitney U tests and similarly showed no differences in improvement or endpoint scores (all *p* ≥ 0.629).

### Primary clinical measures: intervention efficacy

**Fig. 2:**
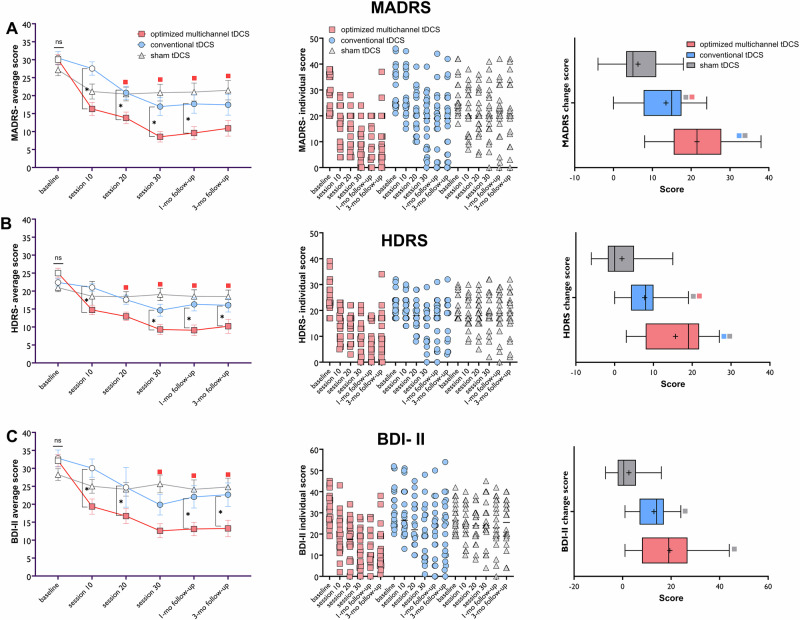
Clinical outcome measures. Depressive symptoms were measured by the Montgomery–Åsberg Depression Rating Scale (**A**), Hamilton Depression Rating Scale (**B**), and Beck Depression Inventory (**C**) before the intervention, at weeks 2 (session 10), 4 (session 20), 6 (session 30), and up to one- and three-month follow-up. In the left panel, filled symbols indicate significant differences at each time point compared to baseline. Floating squares indicate significant differences vs sham tDCS at each timepoint with color denoting to the active group (here red for multichannel tDCS). Asterisks show the difference between the multichannel vs conventional tDCS groups at respective time points. The middle panel displays a scatter plot of clinical scores for each group across time points. The right panel shows the mean score change from the baseline to the study endpoint (week 6) for the respective measure. Floating squares in each arm indicate significant difference with other color-coded arms. The horizontal bar shows the median, the + shows the mean, the upper and lower boundaries show the 25^th^ and 75^th^ percentiles, respectively, and the whiskers show 1–99 percentiles. All pairwise comparisons are conducted with Bonferroni‑corrected multiple comparisons test, and error bars indicate the standard error of the mean (s.e.m.). *Note*: HDRS Hamilton Depression Rating Scale, MADRS Montgomery–Åsberg Depression Rating Scale, BDI-II Beck Depression Inventory-II, tDCS transcranial direct current stimulation, mo month.

The results of the 3 (optimized multichannel tDCS, conventional tDCS, sham tDCS) × 6 (pre, T1, T2, T3, 1-month follow-up, 3-month follow-up) mixed ANOVA revealed significant main effects of time (*F*_*3.61,205.75*_ = 57.34, *p* < 0.001, *η*p^2^ = 0.50), group (*F*_*2,57*_ = 5.21, *p* = 0.008, *η*p^2^ = 0.15) and group×time interaction (*F*_*7.21,205.75*_ = 7.51, *p* < 0.001, *η*p^2^ = 0.21) on MADRS scores. Bonferroni-corrected post-hoc t-tests for the within-group comparisons (baseline vs post-intervention) show that the MADRS scores significantly decreased from session 10 in the optimized multichannel tDCS (*t* = 4.65, *p* < 0.001) and from session 20 in the conventional tDCS (*t* = 3.26, *p* = 0.006), with no significant changes in the sham group (*p* = 0.216). Compared to the sham group (between-group comparisons), Bonferroni-corrected post-hoc t-tests show that MADRS scores significantly reduced only in the optimized multichannel tDCS at sessions 20, 30, 1-month, and 3-month follow-up assessments (Fig. 2A). Similarly for HDRS scores, a significant main effect of time (*F*_*3.27,186.62*_ = 37.90, *p* < 0.001, *η*p^2^ = 0.39), group (*F*_*2,57*_ = 5.24, *p* = 0.008, *η*p^2^ = 0.15) and group×time interaction (*F*_*6.54,186.62*_ = 9.34, *p* < 0.001, *η*p^2^ = 0.24) were found. Bonferroni-corrected post-hoc t-tests for the within-group comparisons revealed that the scores significantly decreased from session 10 in the optimized multichannel tDCS group (*t* = 4.80, *p* < 0.001) and from session 30 in the conventional tDCS groups (t = 3.62, *p* = 0.002), with no significant changes observed in the sham group (*p* > 0.999). When compared to the sham group, the HDRS scores were significantly lower at sessions 20, 30, 1-month follow-up, and 3-month follow-up in the optimized multichannel tDCS group, and no significant changes in the conventional tDCS vs sham (Fig. 2B). The results of all pairwise comparisons are in the supplementary results.

Additionally, the results of intention‑to‑treat analyses were consistent with completer analyses. At the primary endpoint (session 30), least‑squares mean change in MADRS favored multichannel over sham (sham – multichannel Δ = 14.8 points; 95% CI [10.6, 19.0]; *p* < 0.001) and over conventional (conventional – multichannel Δ = 7.86; 95% CI [3.58, 12.13]; *p* < 0.001). Conventional tDCS also outperformed sham tDCS (sham – conventional Δ = 6.93; 95% CI [2.95, 10.92]; *p* < 0.001). Here, positive Δ indicates higher (worse) scores for the first‑listed arm (i.e., comparator group). HDRS showed a similar pattern with sham − multichannel Δ = 12.81; 95% CI [9.03, 16.58]; *p* < 0.001; conventional − multichannel Δ = 7.21; 95% CI [3.60, 10.83]; *p* < 0.001; and sham − conventional Δ = 5.59; 95% CI [2.37, 8.82]; *p* < 0.001. Responder rates (≥50% reduction, mITT; missing = non‑response) were 68.2% (15/22) for multichannel, 37.5% (9/24) for conventional, and 16.0% (4/25) for sham on MADRS; and 63.6% (14/22) for multichannel, 29.2% (7/24) for conventional, and 8.0% (2/25) for sham on HDRS. ARR and NNT estimates were comparable to the results of complete‑case patients (with endpoint data available). See detailed results in the supplementary content and Tables S6, S7.

### Primary clinical measures: treatment response

From baseline to study endpoint (week 6), the mean MADRS score changes were 6.30 (95% CI, 3.42-9.17) in the sham group, 13.50 (95% CI, 10.44-16.55) in the conventional tDCS group, and 21.50 (95% CI, 17.77-25.22) in the multichannel tDCS group, corresponding to 26.09%, 48% and 71.47% score reductions, respectively. For HDRS, mean changes from baseline to week 6 were 1.90 (95% CI, -0.58-4.38), 7.75 (95% CI, 5.58-9.91), and 15.70 (95% CI, 12.12-19.27) in the sham, conventional, and multichannel tDCS groups, corresponding to 10.22%, 37.29%, and 62.27% score reductions. The MADRS response rates (≥50% symptom reduction) were 20% (4/20), 45% (9/20), and 75% (15/20) in the sham, conventional tDCS, and multichannel tDCS groups, respectively, yielding the Number Needed to Treat (NNTs) of 4 (95% CI, 1.90 to 16.52) and Absolute Risk Reduction (ARR) of 30% (95% CI: 5% to 55%) for conventional tDCS vs. sham and the NNT of 2 (95% CI: 1 to 3) with 60% ARR (95% CI: 35% to 85%) for multichannel tDCS vs. sham. The HDRS response rates were 10% (2/20), 35% (7/20), and 70% (14/20) for the same groups (sham, conventional tDCS, and multichannel tDCS groups, respectively), yielding an ARR of 25% (95% CI: 0.3% to 49.7%) compared with sham, corresponding to an NNT of 4 (95% CI: 2 to 333). Multichannel tDCS demonstrated a larger ARR of 55% (95% CI: 30.3% to 79.7%), yielding an NNT of 2 (95% CI: 1 to 4).

### Secondary clinical measure

A significant main effect of time (*F*_*3.47,197.79*_ = 32.15, *p* < 0.001, *η*p^2^ = 0.36), group (*F*_*2,57*_ = 4.74, *p* = 0.012, *η*p^2^ = 0.14) and group×time interaction (*F*_*6.94,197.79*_ = 6.10, *p* < 0.001, *η*p^2^ = 0.17) were found for the BDI-II scores. Bonferroni-corrected post-hoc t-tests for the within-group comparisons show that the BDI-II scores were significantly reduced in the optimized multichannel tDCS from session 10 (*t* = 3.84, *p* < 0.001) and in the conventional tDCS group from 30 (*t* = 3.90, *p* < 0.001) up to follow-up measurements (Fig. 2C). No significant score reduction was found in the sham group after the intervention. When compared to the sham group, BDI-II scores were significantly decreased only in the optimized multichannel tDCS group at sessions 30, 1-month, and 3-month follow-up assessments, but not in the conventional tDCS group. See supplementary results for pairwise comparison values.

### Cognitive performance

**Fig. 3:**
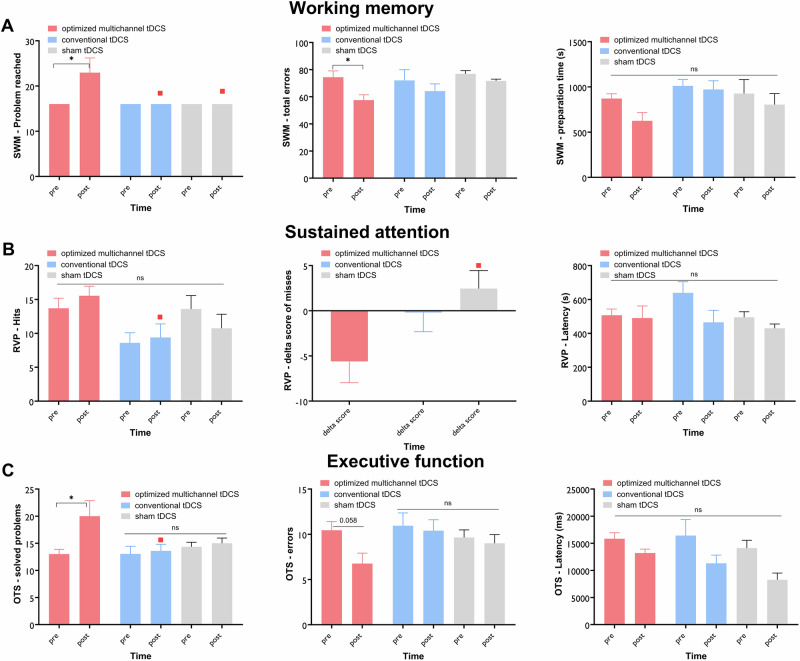
Cognitive control assessment. Cognitive control performance was evaluated before and after 30 sessions of optimized multichannel, conventional, and sham tDCS using the CANTAB neuropsychological test battery for depression, which includes measures of working memory (**A**), sustained attention (**B**), and executive functioning (**C**). Asterisks indicate significant pre-post differences within groups, while colored floating squares indicate between-group differences (in this case, multichannel tDCS vs sham or conventional tDCS) at each time point. Tukey’s multiple comparisons test was used for all pairwise comparisons, and error bars indicate the standard error of the mean (s.e.m.). *Note*: SWM Spatial Working Memory, RVP Rapid Visual Processing, OTS One Touch Stockings of Cambridge, tDCS transcranial direct current stimulation.

Using 3×2 mixed ANOVAs, working memory, sustained attention, and executive functions were assessed before and after the intervention. For working memory, significant main effects of time (*F*_*1,57*_ = 4.84, *p* = 0.032, *ηp2* = 0.080), group (*F*_*2,57*_ = 4.75, *p* = 0.012, *ηp2* = 0.145), and group×time interaction (*F*_*2,57*_ = 4.75, *p* = 0.012, *ηp2* = 0.145) were found on solved problems. Tukey-adjusted multiple comparison tests showed that the optimized multichannel tDCS significantly increased solved problems vs. sham (*p* = 0.025), conventional tDCS (*p* = 0.025), and pre-intervention (*p* < 0.001). A significant time effect (*F*_*1,57*_ = 14.21, *p* < 0.001, *ηp*^*2*^ = 0.200) was observed on SWM performance error, with optimized multichannel tDCS significantly reducing errors (*p* = 0.039) (Tukey-adjusted). No significant effect of time, group, and group×time interaction was found for the SWM preparation time (Fig. 3A).

For sustained attention, significant main effects of group on hits (*F*_*2,57*_ = 3.45, *p* = 0.038, *ηp*^*2*^ = 0.108) and misses (*F*_*2,57*_ = 5.02, *p* = 0.010, *ηp*^*2*^ = 0.150), and a significant group×time interaction on misses (*F*_*2,57*_ = 3.58, *p* = 0.034, *ηp*^*2*^ = 0.112) were found. Tukey-adjusted post-hoc analyses showed no significant pre- to post-intervention changes in misses within any group. However, the multichannel tDCS group had significantly fewer missed trials compared with the sham group. Analysis of delta scores (post- minus pre-intervention) using a univariate ANOVA revealed a significant main effect of group (*F*_*2,57*_ = 3.58, *p* = 0.034, *ηp*^*2*^ = 0.112), with Tukey-adjusted post-hoc test indicating significantly fewer missed trials in the multichannel tDCS group compared to the sham group (*p* = 0.029). For performance speed, no significant main effect of time, group, or group*time interaction was found. (Fig. 3B).

In executive functioning, a significant main effect of time (*F*_*1,57*_ = 7.31, *p* = 0.009, *ηp*^*2*^ = 0.114) and a group×time interaction (*F*_*2,57*_ = 4.45, *p* = 0.016, *ηp*^*2*^ = 0.135) were found for solved problems. A main effect of time also emerged for errors before correct response (*F*_*1,57*_ = 7.66, *p* = 0.008, *ηp*^*2*^ = 0.119) and mean time to correct response (latency) (*F*_*1,57*_ = 15.32, *p* < 0.001, *ηp*^*2*^ = 0.212). Tukey-adjusted post-hoc t-tests revealed that only optimized multichannel tDCS significantly increased solved problems (*p* = 0.004). Error reduction was numerically largest in the optimized multichannel tDCS (35.3% decrease) compared to conventional (-5.02%) and sham tDCS (-6.73%), though differences were not significant (Fig. 3C).

Exploratory Pearson correlations showed that pre–post changes in cognitive performance (SWM errors, RVP misses, and OTS solved problems), and post-intervention cognitive scores, were not significantly associated with symptom improvement at the study endpoint in active tDCS arms (all *p* ≥ 0.166; see supplementary results).

### Functional connectivity

**Fig. 4:**
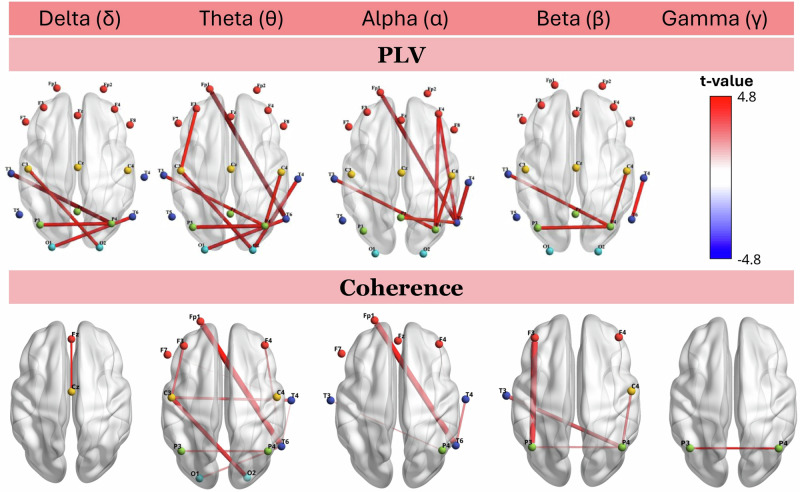
Functional connectivity. Whole-brain functional connectivity after 30 sessions of optimized multichannel tDCS compared to sham tDCS. PLV and coherence values were used to quantify functional connectivity changes in the brain related to cognitive impairment and emotional regulation. No significant post-intervention differences were observed between conventional and sham tDCS. Red edges represent an increase in functional connectivity between nodes. Node colors denote anatomical regions- Frontal (Red), Central (Yellow), Parietal (Green), Occipital (Cyan), and Temporal (Dark Blue). The color bar indicates *t*-values from permutation statistics (red: significant increase in connectivity).

Brain functional connectivity changes before and after the intervention with PLV and coherence. Significant changes were only observed in the post-intervention connectivity of the optimized multichannel tDCS compared to the sham tDCS. Specifically, theta, alpha, and beta PLV values significantly increased in the left frontal-right temporal and left and right occipital regions following optimized multichannel tDCS compared to sham tDCS. For coherence, significant increases were observed in theta, alpha, and beta coherence values after the optimized multichannel tDCS across the left frontal and right temporal regions, as well as for prefrontal-central coherence (Fig. 4). A complementary case–control analysis of the same baseline EEG dataset against demographically matched healthy participants reported network‑level abnormalities at baseline in MDD, pre‑ to post‑intervention connectivity increases in responders that correlated with symptom improvement, and significantly higher post‑treatment connectivity after optimized multichannel tDCS vs conventional and sham tDCS [46]. Detailed directed‑connectivity, graph‑theoretical, and machine‑learning results are reported in that companion article [46].

## Discussion

In this randomized-controlled trial, we evaluated the safety, feasibility, and efficacy of model-optimized multichannel tDCS with 4 mA intensity compared with the conventional 2 mA and sham tDCS for the treatment of MDD. We also assessed cognitive performance and EEG-based functional connectivity changes after the intervention. Both optimized multichannel and conventional tDCS, but not the sham arm, alleviated depressive symptoms after the intervention (at week 6), and these improvements were maintained for up to three months post-intervention. In comparison to the sham, only multichannel tDCS alleviated depressive symptoms. Therapeutic effects in this group emerged as early as week 2 in the, and observed effects were significantly larger than conventional tDCS. Furthermore, the optimized multichannel 4 mA tDCS improved performance in cognitive tasks and increased fronto‑temporal and prefrontal–central functional connectivity following the intervention. Both active interventions resulted in a higher level of reported side effects that were not related to clinical outcomes.

### Safety, feasibility, and treatment adherence

The optimized multichannel protocol delivered a total injected current of 4 mA per polarity (precisely 3.99 mA), which is generally considered safe when applied under established tDCS safety procedures [55, 56]. Consistent with this, no serious adverse events occurred in the present trial, and stimulation was well tolerated across groups. The multichannel montage included multiple smaller electrodes with per-electrode currents ranging from 0.62 to 1.65 mA (current densities 0.20–0.53 mA/cm²), which remained within commonly used safety limits for clinical research protocols [38, 57]. Participants in the multichannel arm reported more frequent and more intense cutaneous sensations (notably itching, tingling, and burning) than the conventional and sham groups, which is unsurprising given the higher current density and multi-electrode configuration [58]. This group reported less fatigue, suggesting a potential activating effect of multichannel tDCS. Importantly, however, the frequency and average reported side effects in the multichannel tDCS group had no significant impact on observed clinical outcomes.

From a feasibility perspective, adherence was acceptable across arms, and attrition patterns were informative. Most dropouts occurred in the conventional and sham groups during early sessions, whereas fewer participants discontinued in the multichannel arm. One possible explanation is that some participants in the multichannel group experienced earlier symptomatic relief and related functional improvements (e.g., mood or sleep-related benefits), which may have increased motivation to continue despite stronger stimulation-related sensations. Overall, these findings support the feasibility of delivering a high-dose, model-optimized multichannel tDCS protocol in a multi-session clinical trial setting, while underscoring the importance of optimizing comfort (e.g., electrode preparation and tolerability management) when higher current density montages are used.

### Blinding efficacy and expectancy effects

The optimized multichannel group showed evidence of unblinding, whereas the conventional group was consistent with chance-level guessing, and the sham group showed opposite guessing. Because MADRS and HDRS are symptom rating scales, differential unblinding raises the possibility that expectancy effects contributed to the larger observed symptom improvement in the multichannel arm. A plausible contributor is the multichannel protocol’s more salient sensory profile (e.g., more frequent or intense itching, tingling, and skin redness), which may function as an “active placebo”, facilitating correct identification of active stimulation. Importantly, however, exploratory analyses found no association between guessed intensity and improvement within that arm, and mean side‑effect ratings were not correlated with HDRS/MADRS change. Nevertheless, expectancy effects cannot be fully excluded. These findings are methodologically important for the field. Previous research occasionally indicates that standard sham protocols may fail to fully mask active stimulation [59, 60] while recent high-definition/high-dose studies show that effective blinding at currents up to 4 mA can be achieved with tailored sham designs (e.g., bimodal ramping) [56]. Overall, our results emphasize that 4 mA multichannel protocols—especially those with higher current density and multi-electrode configurations—pose significant blinding challenges in multi-session clinical trials.

### Intervention efficacy

The therapeutic effects of tDCS in MDD are supported in previous [27, 28] and recent robust studies [22, 61], yet its clinical efficacy is not established, and studies with negative and/or placebo effect [62] have questioned tDCS efficacy. Comparing the clinical efficacy and effect size of this study to recent studies is, nevertheless, informative. One of the largest tDCS studies for depression treatment investigated the efficacy of conventional home-use tDCS [22]. In this study, a conventional tDCS protocol similar to ours significantly reduced depressive symptoms from baseline to week 10, with a mean reduction of 9.41 HDRS points and 11.31 MADRS points in the active tDCS compared to 7.14 HDRS and 7.74 MADRS points for sham treatment. In our study, we observed a mean reduction of 7.75 HDRS points and 13.50 MADRS points in the active conventional tDCS compared to 1.90 HDRS and 6.30 MADRS points for sham treatment. This shows a comparable MDD symptom reduction following the conventional bipolar tDCS in our study to Woodham et al.’s results. A mean reduction of 15.70 HDRS points and 21.50 MADRS points in the optimized multichannel tDCS demonstrates a greater clinical efficacy compared to both conventional and sham tDCS in the compared studies. Interestingly, it shows a similar symptom reduction to a pilot study with multichannel tDCS (n = 35) [63] that reported a mean reduction of 19.8 MADRS score.

The active conventional tDCS protocols’ overall efficacy aligns with recent meta-analyses, indicating that a 2-mA stimulation current during a 30-minute tDCS session can enhance intervention effectiveness [64]. Additionally, our findings support greater efficacy when this intervention is applied using an optimized multichannel configuration. This can be explained from a neurophysiological perspective. The hallmark finding of neuroimaging studies refers to a lateral hypoconnectivity in the prefrontal cortex (especially in the left DLPFC) [65, 66], which is the rationale for applying the excitatory left inhibitory right DLPFC stimulation in depression [20, 67]. Computational modeling of the optimized multichannel tDCS delivered an E-field distribution closely matching the target map for enhanced left DLPFC excitation and right DLPFC inhibition. This optimization yielded a substantially improved fit (0.17 NERNI value for multichannel protocol vs. 0.018 for the bipolar protocol) that might correspond with the larger clinical effect sizes and cognitive enhancement observed in the optimized multichannel tDCS group. This protocol allows a higher total injected current (precisely 3.99 mA), compared to the conventional bipolar montage (2 mA) and more effective modulation of the target regions. In a template head model, the mean En (normal component of the E-field) within the target ROI was ≈1.3–1.4 times greater for the multichannel montage than for the conventional bipolar montage, indicating a higher modeled dose on target.

This finding is consistent with prior work showing that both target-map fit and E-field dose correlate with stimulation effects [68]. Moreover, it suggests that the superior clinical and procognitive outcomes observed with the multichannel configuration may reflect more effective field shaping, increased E-field dose at the target site, or a combination of both, as the two approaches differed in spatial targeting and delivered dose. Nevertheless, future studies using subject-specific forward models and current-balanced comparisons are needed to distinguish montage-shape from dose effects. In line with this are the improved cognitive control functions and enhanced functional connectivity (increases in PLV and coherence in frontotemporal regions), which were observed only after the optimized multichannel vs the conventional sham tDCS protocols and are in line with reduced biomarkers of depression symptoms [10, 11]. These cognitive deficits are key symptoms of MDD, and their improvement, alongside clinical symptoms, indicates that the optimized tDCS intervention improved both mood and cognitive deficits in MDD, which are interconnected [69, 70], and demonstrate its treatment-specific effects at clinical and cognitive levels. The PLV/coherence findings converge with a companion EEG report based on the same dataset, which found reduced baseline connectivity in MDD vs. healthy controls, responder‑linked connectivity increases, and significantly higher post‑treatment connectivity after optimized multichannel tDCS than after conventional and sham stimulation [46].

### Optimized and personalized tDCS: future directions

Optimizing and personalizing transcranial electrical stimulation is a timely and trending topic in current psychiatry and neuroscience [23, 26, 71]. Advances in computational modeling and neuroimaging enable precise tDCS protocols, surpassing conventional bipolar montages by accounting for inter-individual variability in anatomy, conductivity, and connectivity. Yet critical considerations remain regarding efficacy and feasibility. While personalized tDCS holds theoretical promise, its enhanced clinical efficacy requires validation through large-scale, rigorously designed RCTs. Even if proven superior to conventional tES, feasibility poses a significant challenge. The reliance on individualized MRI-based models limits scalability in many clinical and research settings due to high costs and the need for specialized expertise [71]. A promising alternative is model-optimized stimulation using representative head models, which offers a more feasible solution. Supported by a pilot study [63] and our own research, this approach has demonstrated effectiveness in optimizing stimulation at both individual and group levels, which can enhance the accessibility and scalability of model-driven tES protocols across diverse clinical and research settings, specifically in neuropsychiatric conditions marked with similar prefrontal abnormalities and cognitive/emotional deficits [72–74]. It is also of note that personalization efforts often focus on anatomical factors while overlooking dynamic domains such as neurobiological state (e.g., EEG/MEG-guided frequency matching), temporal/neurochemical modulators (e.g., circadian timing or hormonal influences), and mental state/trait (e.g., baseline cognitive performance or anxiety levels) [23]. Integrating these through multiparameter approaches, including closed-loop systems that adjust parameters in real-time based on neurobiological signals, could further reduce variability and enhance efficacy.

### Limitations and strengths

Limitations of this study include the inability to assess cognitive functions and resting-EEG during follow-up assessments due to COVID-19 constraints. In addition, resting-state EEG was recorded with a relatively low-density montage (19 scalp channels), which limits spatial resolution and the ability to precisely characterize network-level connectivity and spectral biomarkers; therefore, subtle effects in measures such as PSD or coherence may have been harder to detect. Next, the reported tDCS side effects were more intense in the optimized multichannel protocol, which is unsurprising given that the multichannel configuration tends to produce stronger perceived sensations due to increased current density. Although reported tDCS side effects were not similar across groups, the parallel group design rules out patients’ awareness of other stimulation arms. Moreover, the reported side effects did not correlate with clinical outcome measures. Our study strengths, on the other hand, include careful monitoring of symptom changes across 6 time-points, assessment of cognitive functions and functional connectivity changes in addition to clinical assessment, and a relatively large sample size, compared to other tDCS trials [67].

## Conclusion

To conclude, the optimized multichannel tDCS was found to be safe with superior clinical efficacy for treating MDD together with procognitive effects for the patients, compared to conventional and sham tDCS. This study highlights the importance of an optimized stimulation protocol to effectively target cortical regions in MDD and provides promising evidence for this practically feasible intervention, in contrast to an individual-MRI-driven optimized intervention. This finding adds further support to the efficacy, safety, and feasibility of DLPFC tDCS as an effective intervention for MDD treatment, not only in remote home-use conventional application [22], but also in the innovative optimized modality with home-use potential [63]. These findings also underscore the relevance of session frequency in assessing clinical efficacy, particularly in the conventional tDCS protocol, which showed antidepressant effects only after session 30, and the placebo effect of tDCS, which was not significant in our study, yet noticeable. Further research, ideally with pre-registration of analytic protocols,should directly compare optimized multichannel tDCS with other standard NIBS approaches.

## Supplementary information

supplementary material

## Data Availability

The data used in this trial will be publicly available at OSF upon manuscript publication via: https://osf.io/xm4vq/overview?view_only=8d2e65ed25ec437c911eaecb8c34ae5d
