# Correction: Optimized multichannel 4 mA vs conventional transcranial direct current stimulation for major depressive disorder: A randomized sham-controlled trial

**DOI:** 10.1038/s41380-026-03780-4

**Published:** 2026-07-27

**Authors:** Mohammad Ali Salehinejad, Marzieh Abdi, Mohsen Dadashi, Maryam Habibi, Amir-Homayun Hallajian, Kiomars Sharifi, Ali Khadem, Ricardo Salvador, Giulio Ruffini, Michael A. Nitsche

**Affiliations:** 1 Department of Child and Adolescent Psychiatry, Psychosomatics and Psychotherapy, Medical Faculty, RWTH Aachen University, Aachen, Germany; 2 Department of Psychology and Neurosciences, Leibniz Research Centre for Working Environment and Human Factors, Dortmund, Germany; 3 Department of Clinical Psychology, Faculty of Medicine, Zanjan University of Medical Sciences, Zanjan, Iran; 4 Donders Institute for Brain, Cognition, and Behaviour, Radboud University, Nijmegen, The Netherlands; 5 Department of Information Technology and Electrical Engineering, ETH Zurich, Zurich, Switzerland; 6 Department of Biomedical Engineering, Faculty of Electrical Engineering, K. N. Toosi University of Technology, Tehran, Iran; 7 Neuroelectrics Barcelona, Barcelona, Spain; 8 Bielefeld University, University Hospital OWL, Protestant Hospital of Bethel Foundation, University Clinic of Psychiatry and Psychotherapy, Bielefeld, Germany; 9 German Center for Mental Health, Bochum, Germany

**Keywords:** Depression, Neuroscience

Correction to: *Molecular Psychiatry* https://doi.org/10.1038/s41380-026-03560-0; published online 02 April 2026

Following publication of this article, the authors identified an error in the corresponding authorship information. Mohsen Dadashi was inadvertently omitted as a co-corresponding author. The corresponding authors for this article are Mohammad Ali Salehinejad and Mohsen Dadashi. Correspondence and requests for materials should be addressed to either author. The original article has been corrected.

