# Supplementary material for: Optimized multichannel 4 mA vs conventional transcranial direct current stimulation for major depressive disorder: A randomized sham-controlled trial
Source: Mol Psychiatry. 2026 Apr 2;31(8):4632–44. doi: 10.1038/s41380-026-03560-0 (PMC13364697; doi:10.1038/s41380-026-03560-0)
Supplement: Supplementary file 1 — supplementary material [file 41380_2026_3560_MOESM1_ESM.pdf]

SUPPLEMENTARY METHODS

Changes in the Clinical Trial registered protocol

The original clinical trial was registered in the local national trial registry (IRCT, WHO-affiliated) before the study's inclusion phase. Initially, a stable treatment regimen was defined as being maintained for at least 4 weeks before and throughout the experiment. However, before the actual study inclusion, this criterion was revised to a stable treatment regimen of at least 6 weeks, aligning with previous studies to keep the medication effect more stable. This change in the inclusion criteria is reflected in the second registration of the trial on clinicaltrials.gov. Additionally, the inclusion criterion for age was originally set between 18 and 75 years, but was later updated to 18 to 60 years to ensure a more reasonable age range. This modification did not impact patient inclusion, as all participants were under 60 years of age.

CONSORT diagram of study inclusion and sample size

Figure S1 illustrates the study inclusion procedure. We initially contacted 167 individuals through social media platforms, telephone outreach, and mostly patient referrals, as depicted in the CONSORT diagram of study inclusion. From this pool, participants were screened for eligibility based on predefined inclusion criteria. Of those contacted, some individuals were excluded during the screening process. The exact number of exclusions and the reasons include 4 due to clinical or neurological conditions, 3 due to an Axis II diagnosis, 3 due to not meeting depression eligibility criteria, and 6 declined to participate. For those enrolled, transportation assistance if needed, session reminder, supportive psychosocial communication by trained staff, and expectation management were provided as much as feasible to support treatment adherence. Due to higher-than-expected dropout rates, a sample size re-estimation was conducted during interim analysis to maintain study integrity. This analysis was planned after 50% of the initial target enrollment (n=30 patients) to assess the pooled dropout rate across groups. If the observed dropout rate exceeded the assumed 5% by more than 10% (threshold >15%), the sample size would be adjusted by a blind enroller. At the interim analysis, after 30 patients were enrolled, the observed dropout rate was 20%. Consequently, the recruitment target was revised to 75 patients to ensure at least 57 completers for the planned statistical power of 0.95. Recruitment continued until 71 patients were enrolled, ensuring at least 20 participants remained for the final analysis. Dropout reasons varied by group: personal reasons (n=2, multichannel group), mismatch between treatment expectations,

personal circumstances, and distant patient locations (n=4, conventional group), as well as lack of symptom improvement after an average of 8 sessions and inconsistent session attendance (n=5, sham group).

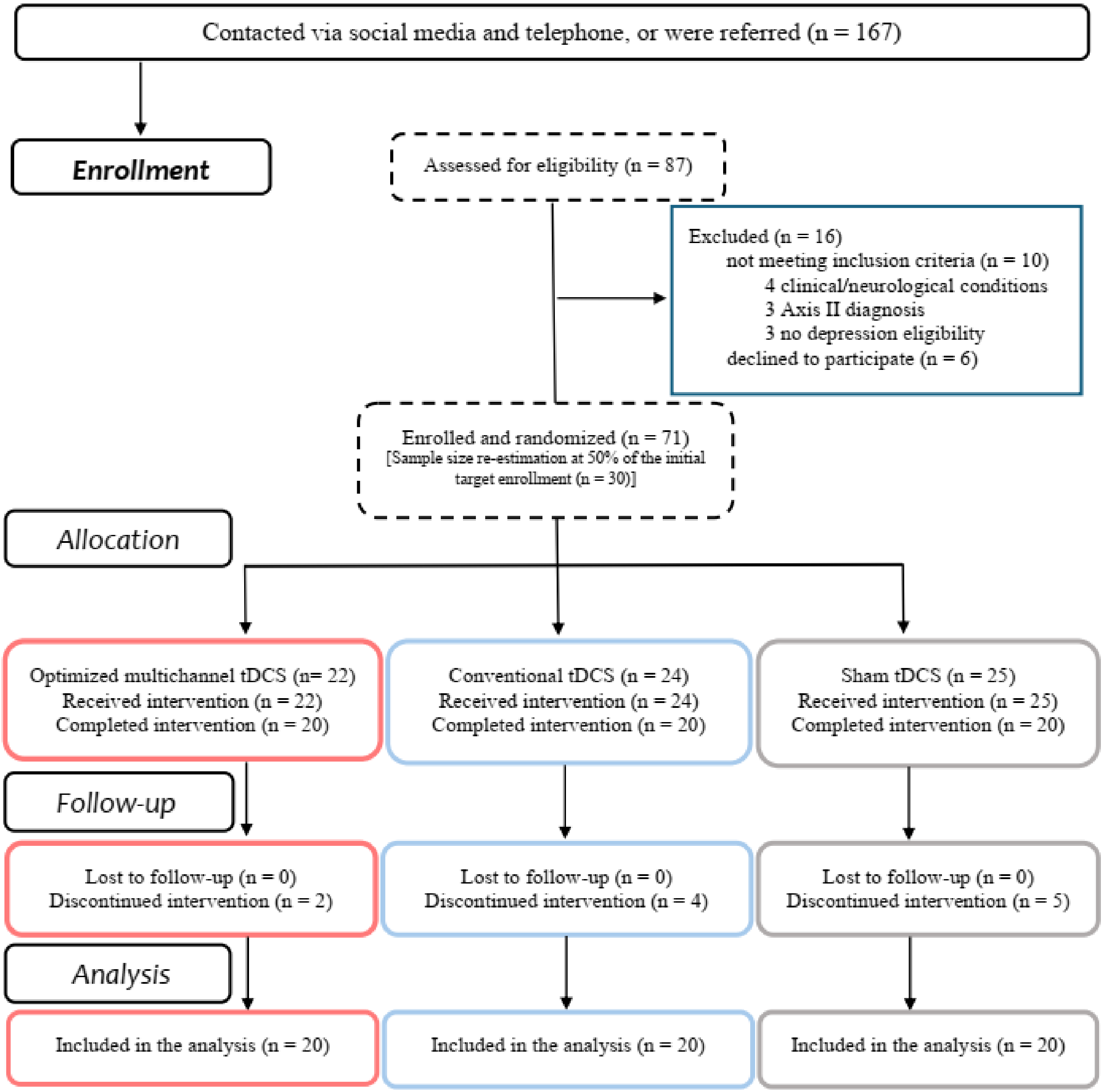

Fig. S1: CONSORT diagram of study inclusion.

Blinding efficacy

Blinding was assessed by asking participants to guess their treatment allocation, with response options of no stimulation (0), mild stimulation (1), or intense stimulation (2). No ”don’t

know" responses were recorded. Bang's Blinding Index (BBI) [1] was used to evaluate the success of blinding in each trial arm. The BBI measures the deviation of participants' guesses from random chance, typically ranging from −1 to 1, where values near 0 indicate successful blinding (random guessing), positive values indicate unblinding toward correct guesses, and negative values indicate guessing opposite to the correct treatment. For this analysis, since both multichannel and conventional arms involved active stimulation, guesses of either mild stimulation (1) or intense stimulation (2) were considered correct for these arms, while a guess of no stimulation (0) was considered correct for the sham arm. This treats the response options into a binary framework: stimulation (1 or 2) versus no stimulation (0). For the multichannel and conventional arms, correct guesses were defined as responses of 1 or 2 (indicating stimulation), while for the sham arm, a correct guess was a response of 0 (no stimulation). The counts of correct guesses were 19/20 for multichannel, 12/20 for conventional, and 4/20 for sham. Table S2 summarizes the BBI results.

OUTCOME MEASURES

Clinical outcome measures

Hamilton Depression Rating Scale (HDRS-17): The Hamilton Depression Rating Scale (HDRS), also known as the Hamilton Scale, is the most widely used clinician-administered questionnaire designed to assess the severity of depression in individuals who have already been diagnosed with the condition. The HDRS is highlighted for its practicality and reliability in evaluating depressive symptoms [2]. The scale typically consists of 17 to 21 items that measure various domains of depression, including mood, guilt, suicide ideation, sleep disturbance, and weight loss. Each item is rated based on the severity of the symptoms, allowing for a comprehensive assessment of an individual's depressive state over time. The HDRS is particularly valued in clinical settings for monitoring treatment efficacy and guiding therapeutic interventions, making it a critical tool in both research and practice for managing depression. A native language version of the test (17 items) with good to excellent psychometric properties was used in this study.

Montgomery–Åsberg Depression Rating Scale (MADRS): The MADRS is another well-known and commonly used tool for evaluating the severity of depressive symptoms, treatment responses, and monitoring patient progress over time [2]. It consists of 10 items that assess various aspects of depression, including various mood disorders such as sadness, pessimism, and inability to experience pleasure [3]. Each item is scored on a scale from 0 to 6, allowing for a total score that reflects the severity of depression. In comparison to other rating scales, the MADRS is particularly valued for its sensitivity to changes in mood and its focus on core depressive symptoms rather than somatic symptoms. A validated native-language version was used in this study.

Beck Depression Inventory (BDI-II): The BDI-II [4] is a 21-item self-report inventory about how the subject has been feeling in the last two weeks. The internal correlational coefficient of the BDI-II is reported between 0.7- 0.92, and the test-retest reliability coefficient is reported as 0.93 at the one-week interval [5]. A native language version of the BDI-II with adequate psychometric properties was used in this study. A score of 20–28 and higher in the BDI-II is indicative of moderate to severe depression. A Cronbach's alpha of 0.94 is reported for the XX language version of the BDI-II.

Cognitive tasks

A neuropsychological test dedicated to depression from the CANTAB computerized test battery [6] was used to assess cognitive deficits in patients before and after the intervention. The battery includes computerized tasks measuring working memory (Spatial Working Memory-SWM), attention (Rapid Visual Processing- RVP), and executive functions (One Touch Stockings of Cambridge- OTS). A detailed description of these measures is provided in the supplementary information. Briefly, the SWM measures working memory and executive functions. Participants are presented with a series of boxes depicted on a screen. Some boxes contain tokens, and the goal is to find and remember the locations of these tokens [7]. The RVP is a sensitive measure of sustained visual attention [8] and presents participants with a white box in the center of the computer screen, inside which digits from 2 to 9 appear in a pseudo-random order at the rate of 100 digits per minute. Participants are asked to detect target sequences of numbers and respond using a press pad. The OTS, a modified version of the Tower of London task, requires participants to rearrange colored balls in vertical columns to match a desired final arrangement in a specified minimum number of moves [7].

Spatial Working Memory-SWM: The SWM task starts with several colored squares (boxes) displayed on the screen. The objective of this task is for the participant to identify one yellow 'token' in each of the boxes by employing a process of elimination and then utilizing them to populate an empty column on the right side of the screen. The number of boxes can be incrementally raised based on the test's difficulty level, with a maximum of 12 boxes shown for participants to examine. To prevent the use of stereotyped search techniques, the color and location of the boxes are varied from one trial to another. We were interested in the Strategy score, average Problems Reached, Total Errors, and preparation time to respond. SWM Strategy is an estimate of the use of this strategy and is obtained by counting the number of times the participant starts a new search with a different box only for six- and eight-box problems. A high score represents poor use of strategy, and a low score corresponds to effective use. SWM Problems Reached (average) quantifies the highest difficulty level attained across trials (i.e., the largest set size of boxes reached and completed) and is reported as the mean across blocks; higher values indicate that the participant progressed to and solved more difficult problems. Lastly, SWM Total Errors is the sum of errors across all trials and includes “between-search errors” and “within-search errors”. Lower values reflect better spatial working memory and more efficient search behavior.

Rapid Visual Processing- RVP: The RVP is a sensitive measure of sustained visual attention [8] and presents participants with a white box in the center of the computer screen, inside which digits from 2 to 9 appear in a pseudo-random order at the rate of 100 digits per minute. Participants are asked to detect target sequences of digits and register responses using the press pad. It takes about 10 min to complete, and the output measure includes latency, which is a good indicator of sustained attentional function.

One Touch Stockings of Cambridge- OTS: The OTS is a test of executive function, based upon the Tower of Hanoi test, and is one subtest of the CANTAB depression battery [8]. During the task, the participant views two displays with three colored balls arranged to resemble stacks held in stockings or socks on a beam. This setup clarifies the 3-D concepts and aligns with the verbal instructions. At the bottom of the screen, there is a row of numbered boxes. The test administrator first demonstrates how to match the pattern in the upper display by moving the balls in the lower display, completing one example that requires a single move. The participant then tackles three additional problems, each requiring two, three, and four moves, respectively. Subsequently, the participant is presented with more problems, needing to mentally calculate the required moves before selecting the corresponding box at the bottom of the screen to indicate their response. Outcome measures include the number of problems solved on first choice, mean latency (speed of response) to first choice, and number of errors.

Methods — Statistical Analysis

We conducted a modified intention-to-treat (mITT) analysis including all randomized participants with baseline assessments (n=71) using a mixed model for repeated measures (MMRM). All randomized participants with baseline data were retained in the mITT set; however, five participants had baseline-only assessments (no post-baseline observations) and therefore contributed no dependent-variable rows to the MMRM estimation, while they were retained for binary endpoint analyses under the missing=non-response rule. Fixed effects were Group (multichannel, conventional, sham), Time (Session 10, Session 20, Session 30, 1-month, and 3-month follow-up), and the Group×Time interaction, with a participant-level random intercept. Models were estimated using (restricted) maximum likelihood with an unstructured covariance matrix (SPSS MIXED; Satterthwaite degrees of freedom). This approach uses all available observations under a Missing-At-Random assumption without ad hoc imputation. Planned contrasts tested between-group differences at each timepoint and within-group change from baseline. Effect sizes are reported as least-squares mean differences with 95% confidence intervals, and we evaluated whether MMRM results were consistent with the complete-case (endpoint-available) analyses. To assess robustness, we further performed sensitivity analyses using (i) multiple imputation (MI; m=50, predictive mean matching) for missing post-baseline scores, and (ii) a control-based "jump-to-reference" pattern-mixture model to assess robustness to Missing-Not-At-Random (MNAR) mechanisms (e.g., assuming dropouts' post-dropout trajectory follows the sham arm).

INTERVENTION

Transcranial direct current stimulation

In conventional tDCS, direct currents were generated by an electrical stimulator and applied through a pair of saline-soaked sponge electrodes (7×5 cm) for 30 minutes (with 30 s ramping up and down) and with an intensity of 2 mA. In sham tDCS, the electrical current was ramped up and down for 30 seconds and then turned off [9]. In the group-optimized multichannel protocols, stimulation was delivered with the StarStim 8 stimulator (Neuroelectrics, Spain) using 7 small electrodes whose positions and currents were determined with the Stimweaver algorithm [10]. This algorithm determines the protocol (currents and electrode positions) that minimizes the weighted difference between the distribution of the normal component of the E-field in the cortical surface (En), to a target En-map. In this particular case, the target En-map was designed to stimulate the left and right DLPFC with desired excitatory and inhibitory effects, respectively. This was achieved by setting the target En as positive (directed into the cortical surface) on the left DLPFC region (here defined as BA46), which leads to an excitatory effect on long pyramidal cells, thus increasing excitability [10, 11]. The opposite was done for the right DLPFC target. The Stimweaver algorithm was run on a template biophysical head model [12], and the current was constrained to a maximum of 2.0 mA per electrode (in absolute value) and 4.0 mA total injected current (here defined as the sum of the currents in all the anodes). Stimulation was delivered on 5 consecutive days for six weeks. In the conventional and sham conditions, anodal and cathodal electrodes were placed over the left DLPFC (F3) and right DLPFC (F4), respectively, using the EEG 10–20 system for electrode positioning. In the multichannel stimulation condition, F3 (1.10 mA, current density ≈0.35 mA/cm²), AF3 (1.24 mA, current density ≈0.40 mA/cm²), and FC6 (1.65 mA, current density ≈0.53 mA/cm²) were stimulated with anodal stimulation, and F4 (-1.28 mA, current density ≈0.41 mA/cm²), AF4 (-1.11 mA, current density ≈0.35 mA/cm²), F8 (-0.62 mA, current density ≈0.20 mA/cm²), and FC5 (-0.97 mA, current density ≈0.31 mA/cm²) were stimulated with cathodal stimulation. The distribution of the component of the E-field normal to the cortical surface induced by the multichannel montage is shown in Figure 1 (main text). This specific configuration of electrodes was expected to result in larger electric fields and more effective modulation of the target regions according to the modeling of different electrode configurations with different intensities and electrode placements. tDCS was applied by independent investigators who were not involved in outcome measure ratings [13]. A side-effect survey was done after each tDCS session [14]. Blinding

efficacy was not explored among patients after the study endpoint to prevent participants' bias from habituation to tDCS-induced sensations over multiple sessions. Yet, after the 2nd follow-up at three months, all patients were asked to choose among 3 options (0, 1, 2) that corresponded to no stimulation, moderate stimulation, and strong stimulation. The patients in the sham group were later assigned to an active tDCS intervention (conventional) after being debriefed about the sham condition, and this phase was not included in the study design.

ELECTROENCEPHALOGRAPHY

EEG preprocessing and data analyses

EEG Preprocessing and Artifact Removal

EEG signals were recorded with a 21-channel EEG device with a sampling rate of 250 Hz, and the included electrodes were Fz, Cz, Pz, C3, T3, C4, T4, Fp1, Fp2, F3, F4, F7, F8, P3, P4, T5, T6, O1, O2, A1, A2. The recordings were obtained in the eyes-open condition for three minutes and then in the eyes-closed condition for three minutes (six minutes in total). To preprocess and remove artifacts from the EEG data, we used Makoto's preprocessing pipeline in the EEGLAB toolbox 2022.1 [15] with MATLAB 2022b (The MathWorks, Natick, MA). Data were resampled to 512 Hz to harmonize with FFT-based pipelines (power-of-two length) and toolbox defaults, high-pass filtered at 1 Hz and re-referenced to an average reference. We used the CleanlineNoise plugin in EEGLAB for line noise removal. We then applied ASR (Artifact Subspace Reconstruction), an automated algorithm that eliminates flatline and noisy channels, low-frequency drifts, and short-time bursts. Any removed channels were interpolated using the spherical interpolation method. Afterward, we visually inspected all the raw data to detect artifact-related parts. To remove non-brain artifacts, we applied Adaptive Mixture ICA (AMICA) to the EEG data to decompose independent components (ICs). We then used the EEGLAB plugin ICLabel to identify brain ICs (with a 'brain' label probability of more than 0.8) from all types of ICs, including Brain, Muscle, Eye, Heart, and others. Finally, we extracted a 60-second segment from the middle part of each preprocessed EEG and exported it to the BRAINSTORM software (version November 2024) for further analysis [16].

Power Spectral Density Analysis

To compute PSD (Power Spectral Density), we used Welch's method [17] on 60-second segments of each condition, with 4-second sliding Hamming windows overlapping at 50%. PSD is a measure of the power of a signal at different frequencies and is commonly used in EEG analysis to identify frequency bands associated with different cognitive processes. We used the [18] pipeline with BRAINSTORM software to perform the analysis. We grouped the PSD values into typical frequency bands of EEG: Theta (5-7 Hz), Alpha (8-12 Hz), Beta (15-29 Hz), and Gamma (30-45 Hz). We computed the PSD absolute values at each frequency bin relative to the total power across the entire frequency spectrum. This allowed us to standardize the PSD values

and compute the relative power for each frequency band. We then obtained relative power values for each electrode, which were used for statistical analysis.

*Functional **connectivity** analysis*

Functional connectivity was assessed using multiple metrics to capture distinct aspects of neural synchronization. The time series from each EEG channel was segmented into 60-second epochs and bandpass filtered into seven frequency bands (delta: 1–4 Hz, theta: 4–8 Hz, alpha 1: 8–10 Hz, alpha 2: 11–13 Hz, beta 1: 13–21 Hz, beta 2: 19–30 Hz, gamma: 30–40 Hz) with finite impulse response filters. The Hilbert transform was then applied to each filtered signal to extract its instantaneous phase. For each pair of channels, connectivity was quantified using four complementary approaches: Phase-Locking Value (PLV) [19], Corrected Imaginary PLV (ciPLV) [20], and Magnitude-Squared Coherence (msCoherence) [21]. PLV measures the consistency of phase differences, ranging from 0 (no synchronization) to 1 (perfect synchronization). ciPLV focuses on the imaginary component of the cross-spectrum, mitigating the effects of volume conduction and common sources. wPLI emphasizes non-zero phase lags, giving greater weight to larger phase differences that are less prone to volume conduction. msCoherence measures the squared magnitude of the cross-spectral density, normalized by the product of each signal's power spectrum, capturing linear correlations at each frequency. All connectivity metrics were computed within the BRAINSTORM framework (Tadel et al., 2011). This multipronged approach provides a more comprehensive evaluation of network-level interactions, reducing the impact of common noise and spurious zero-lag correlations.

EEG Statistical Analysis

To examine the effect of the intervention on relative power spectral density (PSD) values, non-parametric cluster-based permutation t-tests were performed using the Fieldtrip toolbox [22]. The significance level was set at $p<0.05$ with 8,000 iterations. Three distinct analyses were conducted: 1) Within-group analysis comparing post vs pre conditions in each group, 2) Between-group analysis of pre-conditions across experimental groups (sham vs conventional, sham vs Multi-Channel, conventional vs sham), and 3) Between-group analysis of post-conditions across experimental groups. Clusters were defined as regions where two or more adjacent channels exceeded the threshold of $p<0.05$. Functional connectivity was analyzed through three approaches. First, within-group differences between post-test and pre-test were assessed using non-parametric

permutation paired t-tests in Brainstorm [16]. Parameters were set to 8,000 iterations, $p \leq 0.05$ significance level, with FDR correction applied. This analysis targeted each connection within the connectivity matrices. Second, between-group differences in pre-test connectivity values were evaluated using non-parametric permutation independent t-tests with identical parameters. Finally, differences in post-test connectivity values were examined with the same approach. All between-group analyses aimed to identify significant differences in functional connectivity across experimental conditions.

SUPPLEMENTARY RESULTS

Supplementary Results: Intention to Treat Analysis (ITT)

In the modified intention-to-treat set (n=71; multichannel n=22, conventional n=24, sham n=25), 60 participants contributed Session-30 data (n=20 per arm; attrition before Session 30: 2/22, 4/24, 5/25, respectively). All randomized participants with baseline data were retained in the mITT set; however, five sham participants had baseline assessments only (no post-baseline observations) and therefore did not contribute outcome rows to the MMRM estimation, while they were retained for binary endpoint analyses as non-responders under the missing=non-response rule. Using SPSS MIXED (MMRM) with fixed Group, Time, and Group×Time effects, a participant-level random intercept, REML, and an unstructured covariance, the mITT/MMRM findings were consistent with the complete-case (endpoint-available) analyses. At the primary endpoint, baseline-adjusted LSMeans were 22.7 (sham), 15.8 (conventional), and 7.8 (multichannel) for MADRS, and 20.5, 15.0, and 7.6 for HDRS, respectively. Corresponding Session 30 contrasts showed that sham scores were higher (worse) than multichannel on MADRS (Δ = 14.8 points; 95% CI 10.6–19.0; $p<0.001$) and that conventional scores were higher (worse) than multichannel (Δ = 7.86; 95% CI 3.58–12.13; $p<0.001$); sham scores were also higher (worse) than conventional (Δ = 6.93; 95% CI 2.95–10.92; $p=0.00065$). HDRS results mirrored this pattern: sham > multichannel (Δ = 12.81; 95% CI 9.03–16.58; $p<0.001$), conventional > multichannel (Δ = 7.21; 95% CI 3.60–10.83; $p<0.001$), and sham > conventional (Δ = 5.59; 95% CI 2.37–8.82; $p=0.00068$). In a conservative mITT responder analysis (≥50% reduction; missing = non-response), MADRS response rates were 68.2% (multichannel), 37.5% (conventional), and 16.0% (sham), yielding ARR vs sham of +52.2% (95% CI 12.7–77.2%) and +21.5% (−13.5–50.9%) and NNT of 1.92 (95% CI 1.29–7.90) and 4.65 (no finite CI), respectively; HDRS responders were 63.6%, 29.2%, and 8.0%, with ARR vs sham +55.6% (95% CI 18.0–78.0%) and +21.2% (−10.1–46.9%) and NNT 1.80 (95% CI 1.28–5.56) and 4.72 (no finite CI), for multichannel and conventional, respectively. (ARR CIs were computed conservatively, and NNT CIs were obtained by inversion when ARR CIs did not cross 0.) Overall, multichannel tDCS showed large, statistically significant advantages over sham and clear superiority over conventional on both clinician-rated scales at Session 30, and mITT risk metrics (ARR/NNT) aligned with complete-case (endpoint-available) estimates.

Clinical outcome measures: Pairwise comparisons

For the MADRS score, Bonferroni-corrected post-hoc t-tests for the within-group comparisons (BL vs post-intervention) show that in the multichannel tDCS group, the MADRS scores are significantly decreased after sessions 10 (t=4.65, p<0.001), 20 (t=5.50, p<0.001), 30 (t=7.27, p<0.001), 1-month (t=6.92, p<0.001), and 3-month follow-up (t=6.48, p<0.001). In the conventional tDCS group, MADRS scores are significantly decreased after sessions 20 (t=3.26, p=0.006), 30 (t=4.57, p<0.001), 1-month (t=4.31, p<0.001), and 3-month follow-up (t=4.40, p<0.001). No significant changes in the MADRS scores were found for the sham tDCS arm. When compared to the sham arm (between-group comparisons), Bonferroni-corrected post-hoc t-tests show that MADRS scores are significantly lower in the multichannel tDCS group at sessions 20 (t=2.25, p=0.050), 30 (t=4.16, p<0.001), 1-month (t=3.86, p<0.001), and 3-month follow-up (t=3.59, p=0.001), while the conventional tDCS group score drop was not significant with sham at any time points. Additionally, the MADRS symptom reduction in the multichannel tDCS group was significantly higher at sessions 10 (t=3.80, p<0.001), 20 (t=2.37, p=0.018), 30 (t=2.84, p=0.005), and 1-month follow-up (t=2.74, p=0.006) as compared to the conventional tDCS group.

With respect to the HDRS score, Bonferroni-corrected post-hoc t-tests for the within-group comparisons (BL vs post-intervention) show that in the multichannel tDCS group, the HDRS scores are significantly decreased after sessions 10 (t=4.80, p<0.001), 20 (t=5.64, p<0.001), 30 (t=7.35, p<0.001), 1-month (t=7.44, p<0.001), and 3-month follow-up (t=6.92, p<0.001). In the conventional tDCS group, HDRS scores are significantly decreased after sessions 30 (t=3.63, p=0.002), 1-month (t=2.85, p=0.023), and 3-month follow-up (t=2.97, p=0.016). No significant changes in the HDRS scores were found for the sham tDCS arm. When compared to the sham arm (between-group comparisons), Bonferroni-corrected post-hoc t-tests show that HDRS scores are significantly lower in the multichannel tDCS group at sessions 20 (t=2.55, p=0.034), 30 (t=4.56, p<0.001), 1-month (t=4.40, p<0.001), and 3-month follow-up (t=3.86, p<0.001) while the conventional tDCS group's score drop was not significant with sham at any time points. Additionally, the HDRS symptom reduction in the multichannel tDCS group was significantly higher at sessions 10 (t=2.92, p=0.011), 30 (t=2.50, p=0.038), 1-month (t=3.37, p=0.003), and 3-month follow-up (t=2.74, p=0.020) as compared to the conventional tDCS group.

With respect to the BDI-II score, Bonferroni-corrected post-hoc t-tests for the within-group comparisons (BL vs post-intervention) show that in the multichannel tDCS group, the BDI-II scores are significantly decreased after sessions 10 (t=3.84, p<0.001), 20 (t=4.64, p<0.001), 30 (t=5.88, p<0.001), 1-month (t=5.72, p<0.001) and 3-month follow-up (t=5.68, p<0.001). In the conventional tDCS group, BDI-II scores are significantly decreased after sessions 30 (t=3.90, p<0.001), 1-month (t=3.24, p=0.007) and 3-month follow-up (t=3.06, p=0.012). When compared to the sham arm (between-group comparisons), Bonferroni-corrected post-hoc t-tests show that BDI-II scores are significantly lower in the multichannel tDCS group at sessions 30 (t=3.93, p<0.001), 1-month (t=3.33, p=0.003) and 3-month follow-up (t=3.48, p=0.002) while the conventional tDCS group score drop was not significant with sham at any time points. Additionally, the BDI-II symptom reduction in the multichannel tDCS group was significantly higher at sessions 10 (t=3.22, p=0.004), 20 (t=2.41, p=0.049), 1-month (t=2.70, p=0.022) and 3-month follow-up (t=2.83, p=0.015) as compared to the conventional tDCS group.

Reported side effects

Side-effect ratings were analyzed across multichannel, conventional, and sham conditions using a one-way ANOVA, followed by Bonferroni-corrected pairwise Welch's t-tests. ANOVA results revealed significant group differences for itching (F=58.78, p<0.001), tingling (F=21.50, p<0.001), burning (F= 85.48, p<0.001), pain (F=3.29, p=0.044), skin redness (F=8.30, p<0.001), headache (F=19.53, p<0.001), and fatigue (F=11.01, p<0.001). Bonferroni-adjusted pairwise comparisons indicated that multichannel was associated with significantly higher ratings than both conventional and sham for itching (both p<0.001), tingling (both p<0.001), and burning (both p<0.001). Multichannel also showed higher ratings than sham for skin redness (p<0.001) and headache (p≤0.001). Fatigue was significantly lower in the multichannel condition compared to conventional (p<0.001) and sham (p=0.023). Pain differences were modest, with multichannel showing slightly higher ratings than sham (p=0.049), while other pain contrasts were not significant after Bonferroni correction. Pairwise comparisons are presented in Table S4. We additionally computed per-subject adverse-event counts as the number of side-effect categories with non-zero average ratings across all 30 sessions (seven domains: itching, tingling, burning, pain, skin redness, headache, fatigue). The multichannel group showed a higher count (5.05 ± 0.94; range 4–7) than

conventional (2.50 ± 1.15; range 1–5) and sham (2.85 ± 1.04; range 0–4). A composite side-effect burden (sum of the seven mean ratings) was likewise higher in multichannel (12.85 ± 4.06) than conventional (4.90 ± 2.20) and sham (4.35 ± 1.46) (Table S5).

Association between side effects and clinical outcome measures

The association between reported tDCS side effects and clinical measure endpoint (after session 30) was analyzed using Pearson correlational analyses. Side effects included itching, tingling, burning, pain, skin redness, headache, and fatigue (mean ratings across sessions). Clinical outcomes were the HDRS and MADRS scores. Overall, across the multichannel, conventional, and sham groups, mean side-effect severities generally did not significantly correlate with the primary clinical outcome measures.

In the multichannel group, correlations were weak and non-significant for itching (HDRS: r = 0.077, p = 0.746; MADRS: r = 0.053, p = 0.826), tingling (HDRS: r = 0.321, p = 0.167; MADRS: r = 0.327, p = 0.159), burning (HDRS: r = −0.113, p = 0.636; MADRS: r = −0.238, p = 0.313), pain (HDRS: r = 0.092, p = 0.700; MADRS: r = 0.186, p = 0.433), skin redness (HDRS: r = 0.150, p = 0.528; MADRS: r = 0.129, p = 0.588), headache (HDRS: r = −0.053, p = 0.824; MADRS: r = 0.063, p = 0.793), and fatigue (HDRS: r = 0.207, p = 0.381; MADRS: r = 0.202, p = 0.393). In the conventional group, most correlations were also non-significant, including itching (HDRS: r = −0.035, p = 0.884; MADRS: r = 0.116, p = 0.627), skin redness (HDRS: r = 0.233, p = 0.323; MADRS: r = 0.165, p = 0.486), headache (HDRS: r = −0.169, p = 0.476; MADRS: r = −0.153, p = 0.520), and fatigue (HDRS: r = 0.132, p = 0.579; MADRS: r = 0.200, p = 0.399), except for pain, which showed significant moderate correlations (HDRS: r = 0.588, p = 0.006; MADRS: r = 0.618, p = 0.004). Tingling and burning correlations were not computable due to constant values.

In the sham group, all correlations were non-significant, including itching (HDRS: r = 0.096, p = 0.689; MADRS: r = −0.017, p = 0.942), burning (HDRS: r = 0.116, p = 0.627; MADRS: r = 0.182, p = 0.444), skin redness (HDRS: r = −0.132, p = 0.578; MADRS: r = −0.133, p = 0.577), and fatigue (HDRS: r = 0.062, p = 0.795; MADRS: r = 0.154, p = 0.518), with tingling, pain, and headache correlations not computable due to constant values. These findings suggest that, with the

exception of pain in the conventional group, tDCS-related side effects do not significantly predict or influence clinical outcomes in depression as measured by HDRS and MADRS.

We additionally quantified a side-effect *count* per participant, defined as the number of side-effect domains with a non-zero mean rating across all sessions (range 0–7 across the seven domains listed above). We then examined whether this side-effect count is associated with clinical outcome at the primary endpoint (Session 30). Clinical improvement was defined as baseline-to-endpoint change scores: ΔMADRS = MADRSpre − MADRS30 (endpoint) and ΔHDRS = HDRSpre − HDRS30 (endpoint), such that larger positive values indicate greater symptom reduction. Across the full sample (n = 60), side-effect count showed a positive correlation with symptom improvement (ΔMADRS: Pearson $r = 0.379$, $p = 0.0028$; ΔHDRS: Pearson $r = 0.440$, $p = 0.00044$). However, this association was not evident within each treatment arms (all within-arm $p > 0.05$). Because the side-effect count is a discrete variable, we also computed Spearman rank correlations as a robustness check, which yielded the same overall pattern (significant pooled association; non-significant within-arm associations). Finally, to account for potential confounding by treatment arm and baseline severity (i.e., active arms showing both greater side-effect burden and greater improvement), we fit baseline- and group-adjusted linear regression models predicting improvement (ΔMADRS or ΔHDRS) from side-effect count while controlling for baseline symptom severity and group allocation. In these adjusted models, side-effect count was not a significant independent predictor of improvement (both $p \geq 0.50$). Together, these analyses indicate that the pooled correlation between side-effect count and symptom improvement is likely explained by between-group differences rather than a direct relationship between side-effect burden and clinical response.

Association between cognitive changes and clinical outcomes

To examine whether cognitive improvements were related to clinical response, we conducted exploratory correlational analyses between pre–post cognitive change scores and symptom improvement from baseline to the Session 30 endpoint. Clinical improvement was quantified as ΔMADRS = MADRSpre − MADRS30 (endpoint) and ΔHDRS = HDRSpre − HDRS30 (endpoint), such that larger positive values indicate greater symptom reduction. For cognitive outcomes, pre–post change scores were computed for the main cognitive endpoints reported in the manuscript and oriented so that positive values reflect improvement (SWM errors

improvement = SWM errors_pre − SWM errors_post; RVP misses improvement = misses_pre − misses_post; OTS solved improvement = solved_post − solved_pre). Pearson correlations were then calculated between each cognitive change score and ΔMADRS/ΔHDRS within each active treatment arm separately.

In the multichannel arm (n = 20), none of the cognitive change scores were significantly correlated with clinical improvement (SWM errors improvement vs ΔMADRS: $r = -0.260$, $p = 0.268$; vs ΔHDRS: $r = -0.259$, $p = 0.271$; RVP misses improvement vs ΔMADRS: $r = 0.173$, $p = 0.466$; vs ΔHDRS: $r = -0.136$, $p = 0.569$; OTS solved improvement vs ΔMADRS: $r = -0.073$, $p = 0.759$; vs ΔHDRS: $r = -0.322$, $p = 0.166$). Similarly, in the conventional arm (n = 20), no significant associations were observed (SWM errors improvement vs ΔMADRS: $r = 0.002$, $p = 0.993$; vs ΔHDRS: $r = -0.129$, $p = 0.589$; RVP misses improvement vs ΔMADRS: $r = -0.168$, $p = 0.479$; vs ΔHDRS: $r = -0.242$, $p = 0.304$; OTS solved improvement vs ΔMADRS: $r = -0.107$, $p = 0.654$; vs ΔHDRS: $r = -0.226$, $p = 0.337$). Overall, these results indicate that the observed cognitive improvements were not reliably associated with the magnitude of clinical symptom change at the study endpoint in either active stimulation arm.

SUPPLEMENTARY TABLES

Table S1: Means and SDs of clinical outcome measures and neuropsychological test battery performance before the intervention, during, and after the intervention (n = 60 patients with endpoint and follow-up data).

Measure	variable	Time	Group M (SD) multichannel tDCS M (SD)	conventional tDCS M (SD)	sham tDCS M (SD)	*p*-value
**HDRS**	Score	Pre-intervention	25 (6.05)	22.40 (4.46)	20.95 (4.99)	0.063
		Session 10	14.75 (5.91)	21 (4.58)	18.55 (6.06)	
		Session 20	12.95 (5.33)	17.60 (5.72)	18.40 (6.43)	
		Session 30	9.30 (6.39)	14.65 (7.38)	19.05 (7.61)	
		1-month follow-up	9.10 (6.52)	16.30 (8.18)	18.50 (8.26)	
		3-months follow-up	10.20 (8.75)	16.05 (8.41)	18.45 (8.14)	
**BDI-II**	Score	Pre-intervention	32.10 (7.68)	32.80 (10.32)	28.25 (7.39)	0.204
		Session 10	19.35 (9.43)	30.05 (11.36)	25.00 (8.64)	
		Session 20	16.70 (9.32)	24.70 (10.40)	24.15 (8.41)	
		Session 30	12.60 (8.75)	19.85 (12.75)	25.65 (11.09)	
		1-month follow-up	13.10 (8.42)	22.05 (13.88)	24.15 (11.71)	
		3-months follow-up	13.25 (10.21)	22.65 (14.64)	24.80 (11.01)	
**MADRS**	Score	Pre-intervention	30.05 (6.59)	30.45 (8.05)	27.15 (7.27)	0.306
		Session 10	16.30 (8.18)	27.55 (8.28)	21.15 (9.32)	
		Session 20	13.80 (7.26)	20.80 (8.15)	20.45 (8.54)	
		Session 30	8.55 (6.50)	16.95 (11.07)	20.85 (10.41)	
		1-month follow-up	9.60 (7.92)	17.70 (11.55)	21 (11.15)	
		3-months follow-up	10.90 (9.67)	17.45 (12.83)	21.50 (12.06)	
**SWM (working memory)**	Problems reached	Pre-intervention	16 (0.00)	16 (0.00)	16 (0.00)	0.999
		Post-intervention	22.95 (14.26)	16 (0.00)	16 (0.00)	
	Error	Pre-intervention	74.40 (20.81)	72.05 (35.13)	76.90 (10.76)	0.821
		Post-intervention	57.50 (17.62)	64.15 (23.96)	71.70 (5.70)	
	Preparation time (s)	Pre-intervention	912.15 (286.69)	1011.27(316.91)	926.01 (691.63)	0.771
		Post-intervention	811.79 (198.08)	971.54 (430.84)	804.03 (532.65)	
**RVP (sustained attention)**	Hits	Pre-intervention	13.70 (6.64)	8.60 (6.67)	13.60 (8.86)	0.055
		Post-intervention	15.55 (6.29)	9.40 (8.92)	10.75 (9.29)	
	Misses (Δ score (post − pre)	Delta score (pre-post)	-5.60 (10.59)	-0.20 (9.46)	2.45 (8.92)	n/a
	Latency (s)	Pre-intervention	506.81 (155.61)	639.06 (236.31)	494.91 (122.21)	0.067
		Post-intervention	490.50 (302.35)	550.09 (163.37)	430.17 (94.94)	
**OTS (executive function)**	Solved problems	Pre-intervention	13 (3.97)	13.05 (6.30)	14.35 (3.73)	0.606
		Post-intervention	20 (12.78)	13.60 (5.39)	15 (4.31)	
	Error	Pre-intervention	10.45 (4.28)	10.95 (6.30)	9.65 (3.73)	0.700

	Post-intervention	6.76 (5.18)	10.40 (5.39)	9.00 (4.31)	
Latency (ms)	Pre-intervention	15832.17 (4950.26)	16426.21 (13230.57)	14106.51(6471.9)	0.718
	Post-intervention	13202.46 (3269.16)	11300.52 (6800.78)	8272.10 (5517.6)	

Note: tDCS = transcranial Direct Current Stimulation; M = mean; SD = standard deviation; HDRS = Hamilton Depression Rating Scale; BDI-II = Beck Depression Inventory-II; MADRS = Montgomery–Åsberg Depression Rating Scale; SWM = Spatial Working Memory; RVP = Rapid Visual Processing; OTS = One Touch Stocking of Cambridge. *P*-values refer to between-group baseline (pre-intervention) comparisons by one-way ANOVA.

Table S2: Means reported tDCS side effects.

	**Sensation**	**Group**<br>**multichannel tDCS M (SD)**	**conventional tDCS M (SD)**	**sham tDCS M (SD)**	***p*-value**
**Reported side effect**	Itching Sensation	3.25 (1.11)	0.50 (0.51)	1.10 (0.78)	<0.001
	Tingling Sensation	1.90 (1.83)	0.00	0.00	<0.001
	Burning Sensation	2.80 (1.15)	0.00	0.45 (0.51)	<0.001
	Pain at Stimulation Site	0.40 (0.68)	0.30 (0.57)	0.00	0.044
	Skin Redness	2.55 (1.19)	1.70 (1.34)	1.10 (0.78)	0.001
	Headache	1.10 (1.02)	0.10 (0.30)	0.00	<0.001
	Fatigue	0.85 (0.98)	2.30 (1.03)	1.70 (0.92)	<0.001

Values are presented as means ± standard deviation (SD). *Note*: each value represents the average of side effects reported during all 30 tDCS sessions. tDCS = transcranial Direct Current Stimulation; M = Mean; SD = Standard Deviation; ($p \leq 0.05$)

Table S3: Blinding efficacy assessed with Bang's Blinding Index (BBI)

**Group**	**n**	**Correct Guesses**	***p***	**BBI**	**95% CI**
**Multichannel**	20	19	0.95	0.90	(0.53, 0.98)
**Conventional**	20	12	0.60	0.20	(-0.23, 0.56)
**Sham**	20	4	0.20	-0.60	(-0.84, -0.17)

BBI values range from −1 to +1, where values near 0 indicate successful blinding (chance-level guessing), positive values indicate unblinding toward correct identification, and negative values indicate opposite guessing. BBI = Bang's Blinding Index (BBI), CI = confidence interval.

Table S4. Pairwise comparisons of stimulation-related side effects (Bonferroni-corrected)

Side effect	Pair	*t*	*p* (Bonferroni)	Direction
Fatigue	multichannel vs conventional	-4.541	0.00017	higher in conventional
Fatigue	multichannel vs sham	-2.811	0.02335	higher in sham
Skin Redness	multichannel vs sham	4.541	0.00021	higher in multichannel
Tingling	multichannel vs conventional	4.637	0.00054	higher in multichannel
Tingling	multichannel vs sham	4.637	0.00054	higher in multichannel
burning	conventional vs sham	-3.943	0.00262	higher in sham
burning	multichannel vs conventional	10.873	0.0	higher in multichannel
burning	multichannel vs sham	8.343	0.0	higher in multichannel
headache	multichannel vs conventional	4.194	0.00109	higher in multichannel
headache	multichannel vs sham	4.819	0.00036	higher in multichannel
itching	conventional vs sham	-2.854	0.02235	higher in sham
itching	multichannel vs conventional	9.998	0.0	higher in multichannel
itching	multichannel vs sham	7.029	0.0	higher in multichannel
pain	multichannel vs sham	2.629	0.04964	higher in multichannel

Table S5: Per-subject adverse-event counts and overall burden across treatment arms

Descriptive statistics (mean ± SD)					
Group	n	AE_count (mean ± SD)	*F, p*-value	AE_burden (mean ± SD)	*F, p*-value
conventional	20	2.50 ± 1.15	34.85, <0.001	4.90 ± 2.20	57.98, <0.001
multichannel	20	5.05 ± 0.94	-	12.85 ± 4.06	
sham	20	2.85 ± 1.04		4.35 ± 1.46	

Pairwise comparisons (Welch's t-test, Bonferroni-adjusted p-values)				
Metric	Comparison	*t* (Welch)	*p* (Bonferroni)	Direction
AE_count	multichannel vs conventional	7.67	<0.001	higher in multichannel
AE_count	multichannel vs sham	7.00	<0.001	higher in multichannel
AE_count	conventional vs sham	-1.01	0.956	higher in sham
AE_burden	multichannel vs conventional	7.71	<0.001	higher in multichannel
AE_burden	multichannel vs sham	8.82	<0.001	higher in multichannel
AE_burden	conventional vs sham	0.93	1.000	higher in conventional

AE_count = number of side-effect domains with a non-zero mean rating across all 30 sessions (range 0–7; domains: itching, tingling, burning, pain, skin redness, headache, fatigue). AE_burden = sum of the seven per-subject mean ratings across all sessions (higher = greater overall side-effect severity). Values are mean ± SD; F, p-value from one-way ANOVA across groups; pairwise tests use Welch's t with Bonferroni-adjusted *p*-values. Direction indicates the group with the higher mean. Abbreviations: AE = adverse event; SD = standard deviation; n = sample size.

Table S6: Baseline-adjusted least-squares means (LSMeans) across time and Session-30 pairwise contrasts (mITT/MMRM)

Section	Scale	Time / Contrast	Sham	Conventional	Multichannel	Δ	95% CI	*p*
**Baseline-adjusted LSMeans**	**MADRS**	Session 10	22.53	26.07	15.62	—	—	—
		Session 20	21.99	19.88	13.18	—	—	—
		**Session 30**	22.72	15.84	7.80	—	—	—
		1-month	22.97	16.52	8.80	—	—	—
		3-month	23.69	16.14	10.02	—	—	—
	**HDRS**	Session 10	20.32	21.65	13.73	—	—	—
		Session 20	19.65	17.86	11.44	—	—	—
		**Session 30**	20.48	14.95	7.57	—	—	—
		1-month	19.96	16.61	7.33	—	—	—
		3-month	20.02	16.38	8.30	—	—	—
**Session-30 baseline-adjusted contrasts**	**MADRS**	Sham − Multichannel	—	—	—	14.79	10.63–18.95	<0.001
		Conventional − Multichannel	—	—	—	7.86	3.58–12.13	<0.001
		Sham − Conventional	—	—	—	6.93	2.95–10.92	0.00065
	**HDRS**	Sham − Multichannel	—	—	—	12.81	9.03–16.58	<0.001
		Conventional − Multichannel	—	—	—	7.21	3.60–10.83	<0.001

LSMeans are baseline-adjusted values from SPSS MIXED (MMRM) with fixed effects for Group, Time, and Group×Time, a participant-level random intercept, (restricted) maximum likelihood, and an unstructured covariance (mITT set: n=71; randomized multichannel n=22, conventional n=24, sham n=25; 60 provided Session-30 data, n=20 per arm). All randomized participants with baseline data were retained in the mITT set; however, five sham participants had baseline assessments only (no post-baseline observations) and therefore did not contribute outcome rows to the MMRM estimation. Δ denotes the difference in baseline-adjusted LSMeans at Session 30 between the indicated groups. Positive Δ values indicate higher (worse) scores in the first-named group. Two-sided tests; $p<0.05$ considered significant.

Table S7: mITT responder rates (≥50% reduction) with ARR and NNT vs sham

Scale	Group	Randomized (n)	Responders n (%)	ARR vs sham (95% CI)	NNT vs sham (95% CI)
**MADRS**	Sham	25	4 (16.0%)	—	—
**MADRS**	Conventional	24	9 (37.5%)	+21.5% (−13.5% to +50.9%)	4.65 (CI not finite)
**MADRS**	Multichannel	22	15 (68.2%)	+52.2% (+12.7% to +77.2%)	1.92 (1.29 to 7.90)
**HDRS**	Sham	25	2 (8.0%)	—	—
**HDRS**	Conventional	24	7 (29.2%)	+21.2% (−10.1% to +46.9%)	4.72 (CI not finite)

**HDRS**	Multichannel	22	14 (63.6%)	+55.6% (+18.0% to +78.0%)	1.80 (1.28 to 5.56)

Notes: Responder = ≥50% reduction from baseline to Session 30. mITT denominator = all randomized participants; missing Session-30 data counted as non-response. ARR and NNT are calculated vs sham. Abbreviations: mITT = modified intention-to-treat; MADRS = Montgomery–Åsberg Depression Rating Scale; HDRS = Hamilton Depression Rating Scale; ARR = absolute risk reduction; NNT = number needed to treat; CI = confidence interval. ARR 95% CIs were derived conservatively using Wilson score intervals for each group proportion and combining bounds for the risk difference; NNT CIs were obtained by inversion when ARR CIs did not cross 0.

References

1. Bang H, Ni L, Davis CE. Assessment of blinding in clinical trials. *Controlled Clinical Trials* 2004; **25**(2)**:** 143-156.10.1016/j.cct.2003.10.016
2. Boyle GJ, Golden CJ, Stein DJ, Stern Y. The SAGE Handbook of Clinical Neuropsychology: Clinical Neuropsychological Assessment and Diagnosis. 2023**:**
3. Montgomery SA, Åsberg M. A New Depression Scale Designed to be Sensitive to Change. *British Journal of Psychiatry* 1979; **134**(4)**:** 382-389.10.1192/bjp.134.4.382
4. Beck AT, Steer RA, Brown GK. Beck depression inventory-II. *San Antonio* 1996; **78**(2)**:** 490-498
5. Beck AT, Epstein N, Brown G, Steer RA. An inventory for measuring clinical anxiety: psychometric properties. *Journal of consulting and clinical psychology* 1988; **56**(6)**:** 893
6. Sahakian BJ, Owen AM. Computerized assessment in neuropsychiatry using CANTAB: discussion paper. *Journal of the Royal Society of Medicine* 1992; **85**(7)**:** 399-402
7. Sweeney JA, Kmiec JA, Kupfer DJ. Neuropsychologic impairments in bipolar and unipolar mood disorders on the CANTAB neurocognitive battery. *Biological Psychiatry* 2000; **48**(7)**:** 674-684.https://doi.org/10.1016/S0006-3223(00)00910-0
8. Langley C, Sahakian BJ, Robbins TW. Cambridge Neuropsychological Test Automated Battery (CANTAB). *The SAGE Handbook of Clinical Neuropsychology: Clinical Neuropsychological Assessment and Diagnosis* 2023**:** 435
9. Brunoni AR, Schestatsky P, Lotufo PA, Benseñor IM, Fregni F. Comparison of blinding effectiveness between sham tDCS and placebo sertraline in a 6-week major depression randomized clinical trial. *Clinical Neurophysiology* 2014; **125**(2)**:** 298-305.10.1016/j.clinph.2013.07.020
10. Ruffini G, Fox MD, Ripolles O, Miranda PC, Pascual-Leone A. Optimization of multifocal transcranial current stimulation for weighted cortical pattern targeting from realistic modeling of electric fields. *Neuroimage* 2014; **89:** 216-225.https://doi.org/10.1016/j.neuroimage.2013.12.002
11. Galan-Gadea A, Salvador R, Bartolomei F, Wendling F, Ruffini G. Spherical harmonics representation of the steady-state membrane potential shift induced by tDCS in realistic neuron models. *Journal of neural engineering* 2023; **20**(2)**:** 026004.10.1088/1741-2552/acbabd
12. Miranda PC, Mekonnen A, Salvador R, Ruffini G. The electric field in the cortex during transcranial current stimulation. *Neuroimage* 2013; **70:** 48-58.https://doi.org/10.1016/j.neuroimage.2012.12.034
13. Gandiga PC, Hummel FC, Cohen LG. Transcranial DC stimulation (tDCS): A tool for double-blind sham-controlled clinical studies in brain stimulation. *Clinical Neurophysiology* 2006; **117**(4)**:** 845-850.https://doi.org/10.1016/j.clinph.2005.12.003
14. Brunoni AR, Amadera J, Berbel B, Volz MS, Rizzerio BG, Fregni F. A systematic review on reporting and assessment of adverse effects associated with transcranial direct current stimulation. *International Journal of Neuropsychopharmacology* 2011; **14**(8)**:** 1133-1145.10.1017/s1461145710001690
15. Delorme A, Makeig S. EEGLAB: an open source toolbox for analysis of single-trial EEG dynamics including independent component analysis. *Journal of Neuroscience Methods* 2004; **134**(1)**:** 9-21.https://doi.org/10.1016/j.jneumeth.2003.10.009
16. Tadel F, Baillet S, Mosher JC, Pantazis D, Leahy RM. Brainstorm: a user-friendly application for MEG/EEG analysis. *Computational intelligence and neuroscience* 2011; **2011:** 1-13
17. Welch P. The use of fast Fourier transform for the estimation of power spectra: A method based on time averaging over short, modified periodograms. *IEEE Transactions on Audio and Electroacoustics* 1967; **15**(2)**:** 70-73.10.1109/TAU.1967.1161901
18. Niso G, Tadel F, Bock E, Cousineau M, Santos A, Baillet S. Brainstorm pipeline analysis of resting-state data from the open MEG archive. *Frontiers in neuroscience* 2019; **13:** 284

19. Lachaux J-P, Rodriguez E, Martinerie J, Varela FJ. Measuring phase synchrony in brain signals. *Human Brain Mapping* 1999; **8**(4)**:** 194-208.https://doi.org/10.1002/(SICI)1097-0193(1999)8:4<194::AID-HBM4>3.0.CO;2-C
20. Bruña R, Maestú F, Pereda E. Phase locking value revisited: teaching new tricks to an old dog. *J Neural Eng* 2018; **15**(5)**:** 056011.10.1088/1741-2552/aacfe4
21. Nunez PL, Srinivasan R, Westdorp AF, Wijesinghe RS, Tucker DM, Silberstein RB *et al.* EEG coherency. I: Statistics, reference electrode, volume conduction, Laplacians, cortical imaging, and interpretation at multiple scales. *Electroencephalography and Clinical Neurophysiology* 1997; **103**(5)**:** 499-515.10.1016/s0013-4694(97)00066-7
22. Oostenveld R, Fries P, Maris E, Schoffelen J-M. FieldTrip: open source software for advanced analysis of MEG, EEG, and invasive electrophysiological data. *Computational Intelligence and Neuroscience* 2011; **2011**(1)**:** 156869
